# Total chemical synthesis and biophysical properties of a designed soluble 24 kDa amyloid analogue†
†Electronic supplementary information (ESI) available. See DOI: 10.1039/c8sc01790e


**DOI:** 10.1039/c8sc01790e

**Published:** 2018-05-25

**Authors:** Régis Boehringer, Bruno Kieffer, Vladimir Torbeev

**Affiliations:** a Institut de Science et d'Ingénierie Supramoléculaires (ISIS) , International Center for Frontier Research in Chemistry (icFRC) , University of Strasbourg , CNRS (UMR 7006) , Strasbourg , France . Email: torbeev@unistra.fr; b Department of Integrated Structural Biology , Institut de Génétique et de Biologie Moléculaire et Cellulaire (IGBMC) , INSERM (U964) , University of Strasbourg , CNRS (UMR 7104) , Illkirch , France

## Abstract

Discovering molecular probes that specifically recognize distinct amyloid structures is highly important for physiological studies of protein-misfolding diseases as well as for the development of diagnostic reagents and inhibitors of amyloid self-assembly. Here, we demonstrate an approach that allows for identification of *N*-methylated peptides that are specific binders for a particular amyloid fiber subtype (or polymorph). Protein design and chemical synthesis were used to produce covalently tethered amyloid analogues with molecular masses approaching 24 kDa and containing nine copies of an amyloidogenic peptide. Such engineered constructs served as a molecular testing platform to evaluate the aggregation properties and solubility as a function of *N*-methylation pattern. An advantage of the method is the possibility of biophysical characterization of amyloid constructs in solution.

## Abstract 2

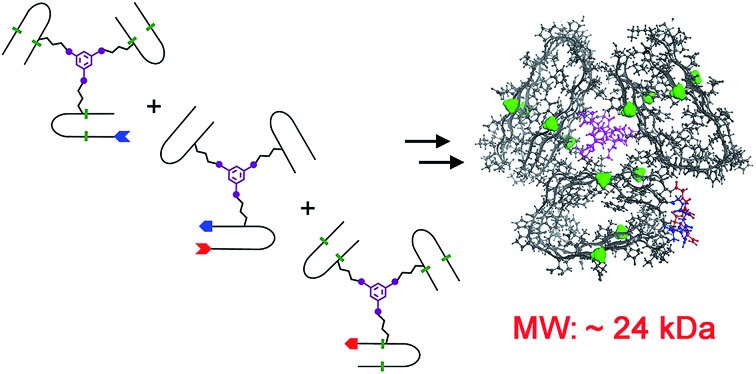
A soluble amyloid analogue was designed and prepared by total chemical synthesis using native chemical ligation.

## 

Protein misfolding diseases are characterized by *in vivo* deposition of insoluble amyloid fibers resulting from aberrant conformational rearrangement and self-assembly of soluble monomeric peptide or protein precursors.^1^ Inhibiting amyloid formation is considered to be a possible therapeutic strategy with some promising results reported for anti-amyloid antibodies.^2^ Increasingly more structural information is available for amyloid fibers with several high-resolution structures recently reported based on solid-state NMR^3^ and cryo-electron microscopy studies.^4^ Remarkably, the same polypeptide sequence can result in amyloids with different structures, *i.e.* distinct polymorphs (also called “amyloid strains”).^5^ Moreover, for Aβ(1–40) and Aβ(1–42) peptides the correlation was established between different amyloid structures and the clinical subtypes characterized by altered rates of progression of Alzheimer's disease.^6^ These results are highly important and will facilitate the development of molecular probes that can specifically recognize the distinct amyloid polymorphs *in vivo* for physiological studies and which can also be elaborated into diagnostic reagents and/or inhibitors.

However, such a task presents significant challenges. The major complications are (i) structural heterogeneity as well as compositional complexity in amyloid self-assembly mechanisms due to the presence of transient oligomers or intermediate protofibers;^7^ and (ii) the insoluble nature of amyloids, which prevents the use of biophysical tools such as solution NMR or isothermal titration calorimetry commonly applied for the analysis of compound binding to biomolecules.

To overcome these complications we considered elaborating a different approach. Starting from already available amyloid structures, protein design and total chemical synthesis can be applied in tandem to produce structural analogues of amyloids that are compositionally well-defined (as opposed to a mixture of oligomers) and behave as soluble species. Amyloids are multimeric supramolecular assemblies formed by amyloidogenic peptide or protein building blocks; hence, our goal here is to apply covalent tethering of several such peptide subunits into a construct in which the structure of a given amyloid polymorph is stabilized. In addition, modifications that prevent this construct from uncontrolled supramolecular polymerization need to be introduced, thus making it soluble. As a result, such amyloid analogues can be studied using various solution-phase biophysical methods.

In this study, the design of a covalently tethered construct is inspired by the structural model of the Aβ(1–40) amyloid with a 3-fold symmetric arrangement of peptide subunits.^8^ In this structure, three copies of Aβ(1–40) adopt a β-arch (β-strand-turn-β-strand) conformation in which β-strands interact *via* amino acid side chains and not through backbone hydrogen bonds. The amyloidogenic [20–41]-segment of human β2-microglobulin ([20–41]β2m) adopts a β-arch conformation similar to Aβ(1–40)^9^ but it represents a more tractable system to explore methodologies of chemical synthesis towards various multimeric constructs. Previously, we demonstrated that [20–41]β2m covalently tethered into a “covalent trimer” aggregates into morphologically distinct amyloids.^10^ In this study, we engineered a covalently tethered oligomer composed of three units of the [20–41]β2m covalent trimer. As a result, the final construct comprises nine copies of the progenitor [20–41]β2m peptide in total (Fig. 1).

**Fig. 1:**
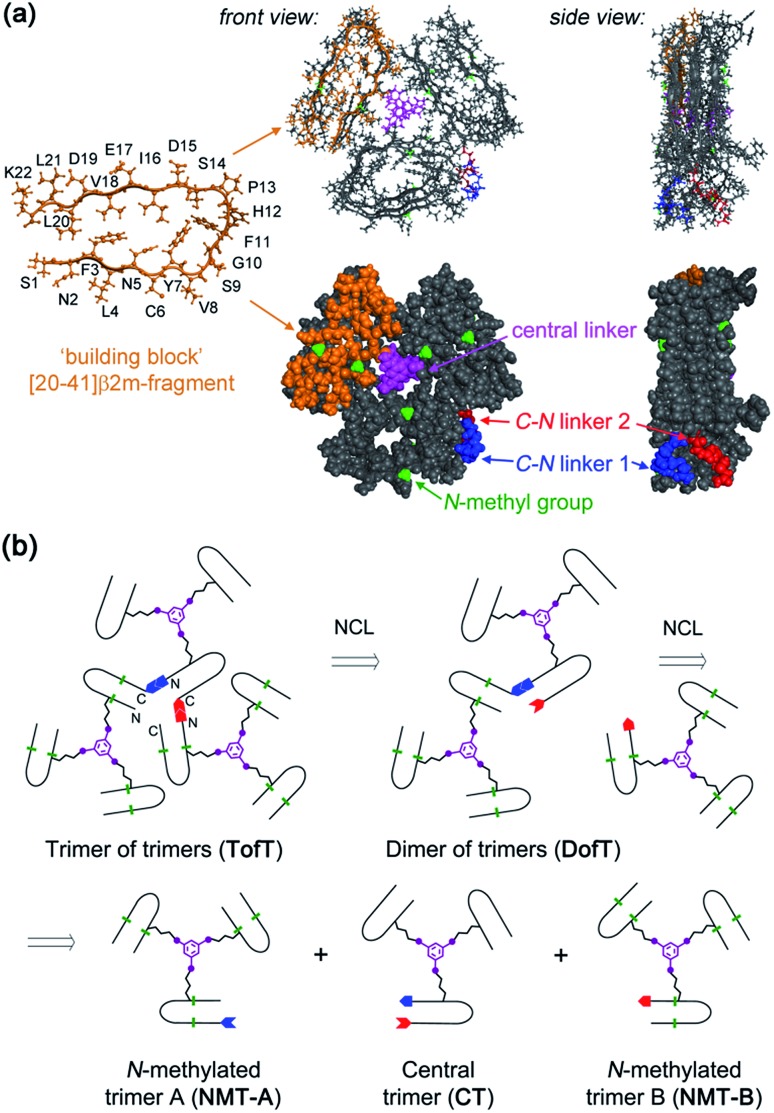
Designed covalently tethered amyloid analogue. (a) Molecular model in ball-and-stick (top) and space-filling (bottom) representations. The construct is composed of nine subunits derived from the amyloidogenic peptide (in orange) adopting a β-arch conformation (based on PDB ID: 2E8D; amino acid residues are abbreviated as one-letter symbols). Each such subunit is joined *via* a central linker (in magenta) to form covalent trimers. Three covalent trimers are assembled with the help of two C–N linkers (in blue and red) connecting the C- and N-termini of the corresponding building blocks to form parallel β-sheets. The covalent trimers that are exposed to solvent are modified by *N*-methylation (in green) to prevent their aggregation into amyloids and to ensure a monomeric state of the species. (b) Retrosynthetic analysis for total chemical synthesis of the designed construct by sequential native chemical ligation (NCL) of three different covalent trimers.

The design of this complex peptide architecture was realized with the help of molecular modelling taking into account the structural properties of the [20–41]β2m covalent trimer.^10^ We envisaged three copies of covalent trimer [20–41]β2m stacked in a parallel, in-register orientation, so that the internal covalent trimer being “sandwiched” by two external trimers has surroundings identical to those found in a complete amyloid fiber (Fig. 1). The desired arrangement of three covalent trimers is enabled by conformationally flexible tetra-glycine chains used as covalent tethers connecting the C- and N-termini of the corresponding peptide building blocks (“C–N linkers”). To prevent uncontrolled spontaneous fibrillization, a subset of solvent exposed amides in the external trimers is modified by *N*-methylation with the purpose of disrupting hydrogen bonding within β-sheets necessary for amyloid propagation (Fig. 1a).

Retrosynthetic analysis towards total synthesis of the designed peptide construct is outlined in Fig. 1b. The synthetic target represents a polypeptide with a non-linear topology. The sequential tethering of the covalent trimers is carried out by native chemical ligation;^11^ therefore, one of the peptide subunits in each covalent trimer is modified with the required functionalities such as a Cys-residue at the N-terminus and an ^α^hydrazide moiety at the C-terminus. ^α^Hydrazides can efficiently be converted into ^α^thioesters.^12^ Cys is introduced in place of one of the Gly-residues in the tetra-glycine linker (hence the final linker sequence is Cys-Gly-Gly-Gly). As a result, three non-identical covalent trimers need to be prepared for the assembly of the desired covalently tethered construct starting from six modified [20–41]β2m peptides (Fig. 1b and ESI Tables S1–S3†).

In our previous work on covalent trimer [20–41]β2m amyloids, the trivalent linker *N*,*N*′,*N*′′-(benzene-1,3,5-triyl)-tris(acetamide) was used to join three copies of the [20–41]β2m peptide.^10^ The conjugation was based on a nucleophilic displacement of a symmetrically substituted bromoacetamide derivative by a Cys6 residue of the [20–41]β2m. In this work, according to our design objectives, the central linker needs to support the attachment of different peptides that would enable introduction of the additional C–N linkers. Thus, the central core linker was designed to support the synthesis of covalent trimers that are composed of two identical peptides and the one that is distinct. As required, the structure of the new central linker 3,5-bis((allyloxycarbonyl)amino)benzoic acid contains two amines protected by Alloc (allyloxycarbonyl) groups and one reactive carboxylic acid (ESI Fig. S1†). The unprotected carboxylic acid is coupled to the side chain amino-group of ornithine introduced at position 6 of the [20–41]β2m peptide (Fig. 2). Ornithine was chosen because the length of the side chain is optimal and leads to the same number of atoms between the peptide backbone and the aromatic ring of the central linker. The corresponding peptide was synthesized with the side-chain of the ornithine protected by Alloc, which is orthogonal to the side chain protecting groups of the other amino acids used in Fmoc/*t*Bu-based solid phase peptide synthesis (SPPS). It was deprotected at the end of the chain assembly on the solid support and the central core linker was directly conjugated (Fig. 2). In the next step, the primary aromatic amino groups of the central linker were Alloc-deprotected and bromoacetylated. The final step is the cleavage of the modified peptide from the resin and purification by HPLC. The formation of the non-symmetric covalent trimers was then achieved by reaction with the required [20–41]β2m-derived peptides having the Cys-residue at position 6 (Fig. 2 and ESI Fig. S2†).

**Fig. 2:**
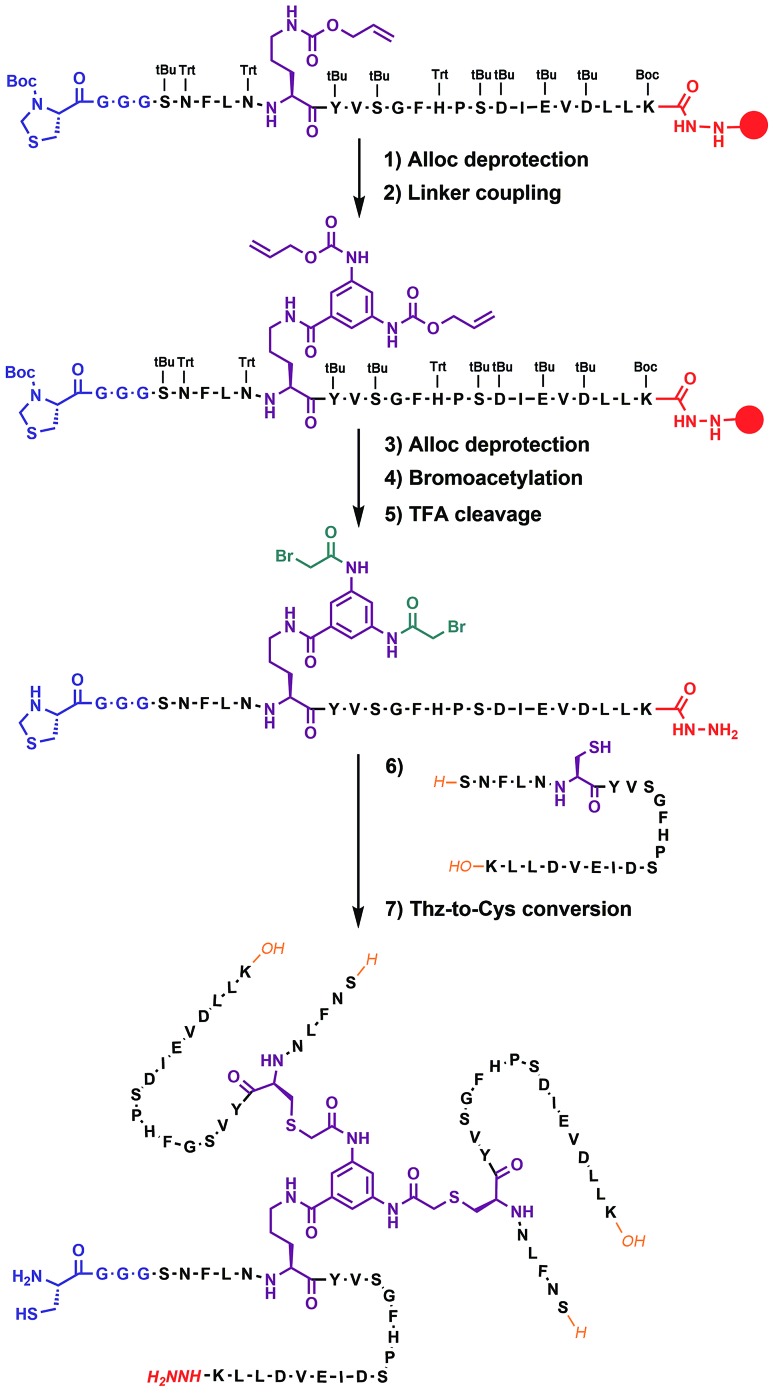
Chemical synthesis of covalent trimer **CT-1** (see also ESI Table S1†) illustrating the chemical steps in the synthesis of non-symmetric covalent trimers. Analytical monitoring of the reaction is provided in ESI Fig. S2.† Amino acid residues are shown as one-letter symbols. ‘H’ and ‘OH’ in orange stand for unmodified amino and carboxy functionalities of the N- and C-termini of the peptide chain, respectively. Red circles represent the polymer resin support. Abbreviations for protecting groups: *t*Bu = *tert*-butyl, Trt = trityl, Boc = *tert*-butyl-oxycarbonyl, and Thz = thiazolidine.

Native chemical ligation was used to join the three non-symmetric covalent trimers in the required order (Fig. 3a and ESI Fig. S3†). *N*-methylated trimer A (**NMT-A**) was obtained as an ^α^thioester and ligated to the central trimer (**CT**). Subsequently, the Cys-residue at the ligation junction was alkylated with 2-bromoacetamide to avoid undesirable Cys-oxidation and the ^α^hydrazide group was converted into an ^α^thioester followed by HPLC purification. Then, the second ligation was performed with *N*-methylated trimer B (**NMT-B**). After subsequent Cys capping with 2-bromoacetamide and HPLC purification, the desired product was obtained.

**Fig. 3:**
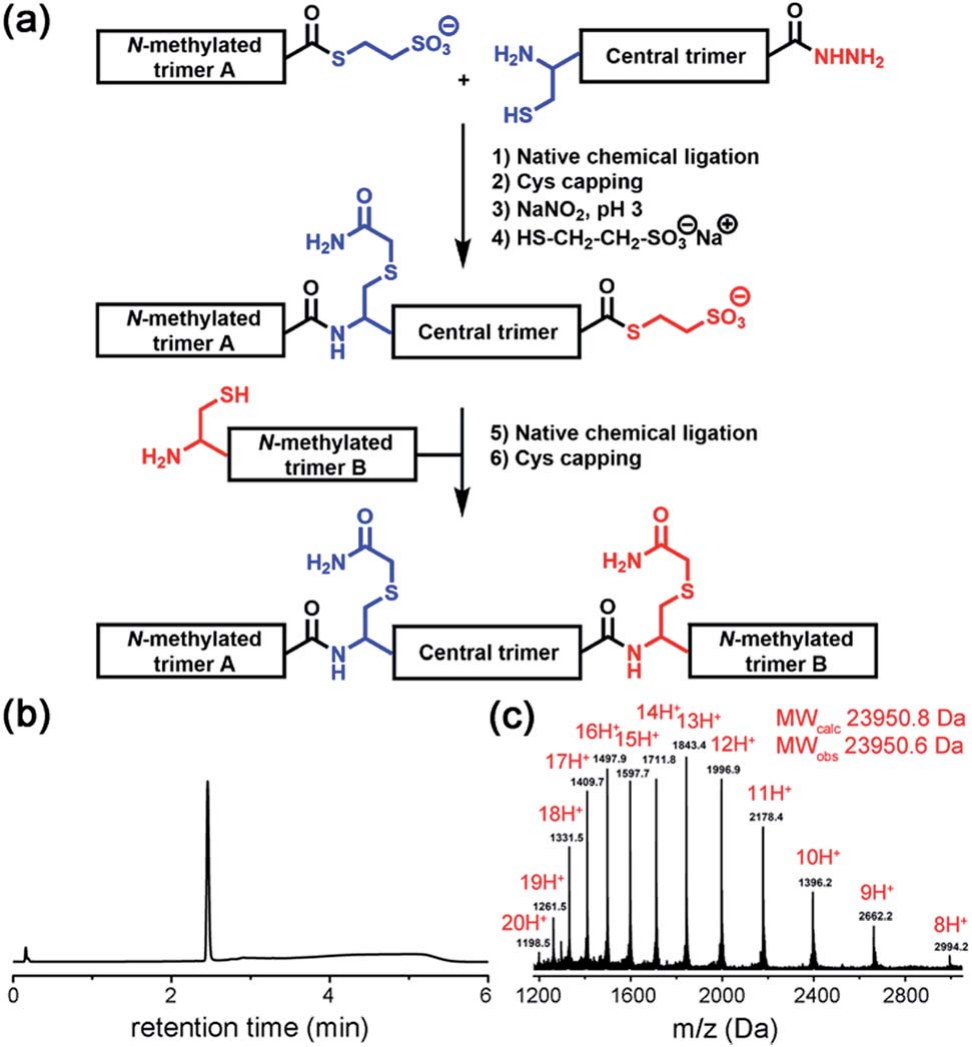
(a) Native chemical ligation was used to join three covalent trimers. (b and c) Analytical HPLC and ESI-MS-Orbitrap analyses of the synthesized **TofT-2**.

Several covalently tethered amyloid analogues were prepared according to this strategy (for more details, yields and analytical characterization, see Fig. 3 and ESI Fig. S4 and S5†). In the first construct **TofT-1** (*trimer of trimers 1*) only one *N*-methyl group per β-arch was introduced into external covalent trimers resulting in six *N*-methyl groups in total (**NMT-A-1**: *N*-Me-Ile16; **NMT-B-1**: *N*-Me-Glu17). In the second variant **TofT-2** (molecular model shown in Fig. 1a), two amino acids per β-arch (twelve *N*-methyl groups in total) were substituted by the corresponding *N*-methylated congeners (**NMT-A-2**: *N*-Me-Asn5 and *N*-Me-Val18; **NMT-B-2**: *N*-Me-Leu4 and *N*-Me-Asp19). Finally, the third construct **TofT-3** was identical to the second, except one of the β-arch peptides in the central trimer was ^15^N-isotope labelled at five residues (Phe3, Val8, Gly10, Ile16 and Leu20).

To characterize the solubility and aggregation state of the synthesized constructs we used size-exclusion chromatography (Fig. 4a). We found that one *N*-methylated amino acid per β-arch in **TofT-1** is not sufficient to prevent the construct from aggregation. Oligomers of **TofT-1** formed instantaneously and upon longer incubation evolved into larger species. TEM analysis confirmed the presence of insoluble aggregates (ESI Fig. S6†). Strikingly, the introduction of two *N*-methylated amino acids per β-arch (one per β-strand) in **TofT-2** was sufficient to stabilize it from aggregation: monomeric species were observed upon prolonged (1 week) incubation (Fig. 4a).

**Fig. 4:**
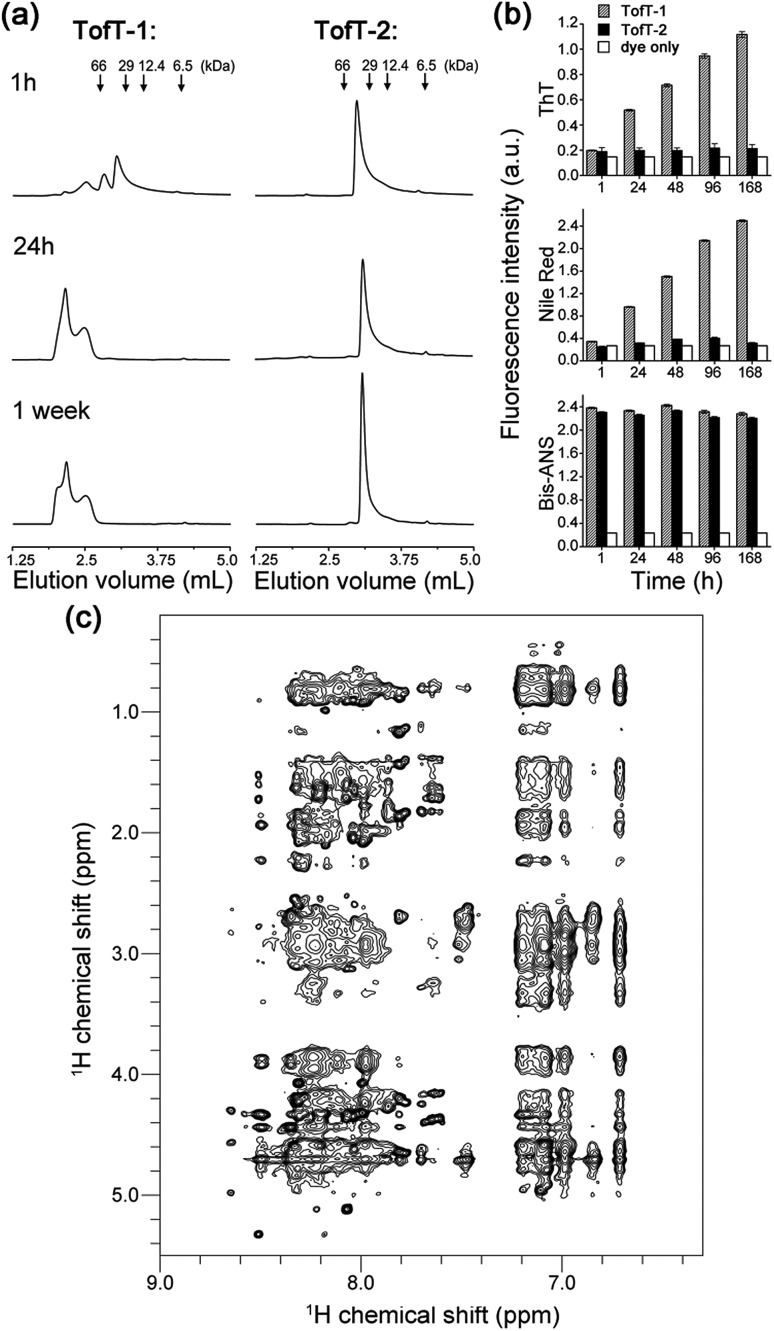
Properties of the synthesized **TofT-1**, **TofT-2** and **TofT-3** constructs. (a) Size-exclusion chromatography of aggregating **TofT-1** and monomeric **TofT-2**. (b) Binding of ThT, bis-ANS and Nile red fluorescent dyes to **TofT-1**, whereas for **TofT-2** only bis-ANS binding was detected. (c) ^1^H-NOESY spectrum recorded for **TofT-3**. See the ESI† for details.

The **TofT-1** and **TofT-2** constructs were tested for their ability to bind fluorescent dyes commonly used for the detection of amyloid fibers or solvent exposed hydrophobic pockets in proteins. We tested dyes such as thioflavin T (ThT), Nile red and bis-ANS. The dyes were added to solutions of covalently tethered constructs (*c* 30 μM) at a concentration of 90 μM giving a protein : fluorescent dye concentration ratio of 1 : 3. **TofT-1** gave strong ThT and Nile red responses compared to a weak signal observed for **TofT-2** (Fig. 4b). ThT and Nile red are typical dyes used to detect the presence of larger aggregates such as amyloids; therefore, these results are in agreement with the enhanced aggregation propensity of **TofT-1** when compared to non-aggregating **TofT-2**. However, with bis-ANS both **TofT-1** and **TofT-2** showed a strong fluorescence response (Fig. 4b). Bis-ANS can serve as a probe for amyloids but it is also known to bind to hydrophobic patches in soluble proteins.^13^ Therefore, efficient binding of bis-ANS to **TofT-2** suggests the presence of solvent exposed hydrophobic pockets.

**TofT-1** and **TofT-2** were also analyzed by ^1^H NMR (ESI Fig. S7†). Very broad and weak signals were present in the spectra of **TofT-1**, which suggests that the major part of the protein aggregated – herein the larger species are not detectable by solution NMR. In contrast, soluble non-aggregating **TofT-2** shows well detectable signals in the amide and aromatic regions. Spectra were also recorded for **TofT-3** (^15^N-labelled version of **TofT-2**) at 25, 30 and 35 °C (ESI Fig. S8†). The dispersion and line-width improve at 35 °C; therefore, subsequent 2D spectra were recorded at this temperature.

The ^1^H-NOESY spectrum of **TofT-3** (Fig. 4c) shows some interesting features: (i) the presence of both sharp and broad resonances; and (ii) a large number of NOE cross-peaks (at ∼7 ppm) presumably corresponding to the aromatic central linker inside a hydrophobic core. A ^1^H–^13^C HSQC spectrum was also recorded, showing well-resolved cross-peaks with a large spread of peak intensities (ESI Fig. S9†). The ^1^H–^15^N HSQC spectrum of ^15^N-labelled **TofT-3** displays five resonances as expected (ESI Fig. S10†). Two of those peaks with ^15^N chemical shifts at 121.5 ppm and 118.5 ppm are of similar and high intensity, while the other three signals at 118.7, 119 and 109.9 ppm are weaker. The latter cross-peak can be assigned to Gly10 based on the distinct chemical shifts for glycines, whereas for the other peaks, unambiguous assignment was not achieved. ^1^H–^15^N relaxation experiments yielded ^15^N *T*_1_ and *T*_2_ relaxation times suggesting a uniform dynamic behaviour of the construct (ESI Fig. S11 and S12†). Furthermore, the *T*_1_/*T*_2_ ratio provides an estimate of the global correlation time of **TofT-3** between 9.8 and 13.2 ns in agreement with the expected correlation time of a protein composed of ∼200 amino acids at 35 °C (11.5 ns is an estimated value from the model of Daragan^14^).

In addition, DOSY (diffusion ordered spectroscopy) experiments support the globular shape of the **TofT-3** species, where the measured diffusion coefficient displays the expected linear dependence when correlated to the variation of viscosity as a function of temperature and corresponds to a molecular weight of ∼20 kDa, consistent with the size of **TofT-3** (ESI Fig. S13†). Overall, NMR data confirm a monomeric form for **TofT-2** and **TofT-3**; however, their structural properties are similar to those of a molten globule rather than a well-structured protein. Binding of bis-ANS is in agreement with this conclusion and was directly confirmed *via* chemical shift changes in the NMR spectrum of **TofT-3** in the presence of bis-ANS (ESI Fig. S14†).

The finding that two methyl groups per β-arch in **TofT-2** are sufficient to prevent it from aggregation has important implications. **TofT-2** (or **TofT-3**) essentially represents a fragment of an amyloid rendered soluble. The non-methylated central trimer **CT** when taken separately corresponds to the basic repeating unit of the amyloid polymorph with a trimeric core. We proved that central trimer **CT** is indeed amyloidogenic; moreover, the morphology of the resulting fibers is similar to that previously determined for covalent trimer [20–41]β2m (ESI Fig. S15†). The two *N*-methylated trimers covalently tethered to **CT** prevent it from aggregation; thus, they can be regarded as amyloid growth inhibitors that recognize and bind at two non-equivalent ends of this minimalistic amyloid building block.

To illustrate the molecular recognition and potency of inhibition of amyloid growth by different *N*-methylated constructs we performed amyloid inhibition assays. By using two monomer peptides, two covalent dimers and two covalent trimers synthesized possessing the same *N*-methylation patterns to prevent aggregation on both sides of the putative amyloid fiber axis as in the **TofT-2** construct, we inspected which of them is the most efficient in inhibiting the growth of non-methylated covalent trimer [20–41]β2m amyloids. As illustrated in Fig. 5, the *N*-methylated trimers are more efficient in suppressing the formation of the respective cross-β structures than the covalent dimers or monomers. This result highlights the significance of structural complementarity in the molecular recognition at the cross-section of amyloid fibers and feasibility for elaborating specific binders to distinct amyloid polymorphs.

**Fig. 5:**
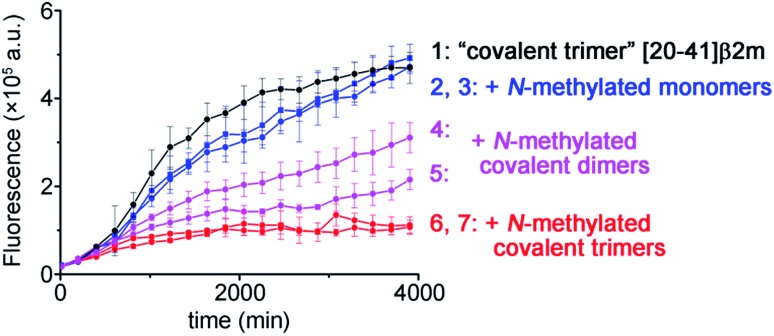
Kinetics of the aggregation of covalent trimer [20–41]β2m monitored by ThT fluorescence specific to the cross-β amyloid structure and the effect of addition of the distinct *N*-methylated peptide inhibitors. Data points are averages of three experiments, and error bars depict the standard deviations. See ESI Fig. S16† for additional experimental information.

## Conclusions

In summary, in this study we proposed a novel approach for studying molecular interactions that involve amyloids. Commonly used random testing of various compounds that can interact with amyloids or influence their growth kinetics does not reveal the molecular details of a compound's interactions with fibers. By designing and synthesizing an amyloid analogue that can be studied in solution by various analytical techniques, we found that the *N*-methylation pattern critically influences the solubility properties of the construct. *N*-methylation is a promising strategy and needs to be explored further, because such peptides can be designed to be fully complementary to an amyloidogenic peptide (or protein) target and are, furthermore, membrane-permeable and protease-resistant.^15^ Thus, our proof-of-principle study outlines a conceptual workflow for future design and evaluation of peptide-based binders specific to a particular amyloid polymorph, which can then be developed into diagnostic reagents or inhibitors – an especially pressing and unresolved task in Alzheimer's disease research.

## Conflicts of interest

There are no conflicts to declare.

## Supplementary Material

Supplementary information
