# Supplementary material for: Total chemical synthesis and biophysical properties of a designed soluble 24 kDa amyloid analogue
Source: Chem Sci. 2018 May 25;9(25):5594–9. doi: 10.1039/c8sc01790e (PMC6049524; doi:10.1039/c8sc01790e)
Supplement: Supplementary file 1 [file SC-009-C8SC01790E-s001.pdf]

This journal is © The Royal Society of Chemistry 2018

Total chemical synthesis and biophysical properties of a designed soluble 24 kDa amyloid analogue

Régis Boehringer, Bruno Kieffer and Vladimir Torbeev*

[*email: *torbeev@unistra.fr*]

<u>Contents:</u>

Materials and methods	p.2-5
Chemical synthesis	p.5-11
Tables S1-S3	p.12-18
Figures S1-S16	p.19-35
References	p.36

Materials and methods

Reagents: All solvents, chemicals, and reagents were purchased from commercial sources and used without further purification. Coupling reagents, Fmoc-α-*L*-amino acids, Fmoc-*N*-methyl-α-*L*-amino acids and resins for SPPS (solid-phase peptide synthesis) were purchased from Iris Biotech (Marktredwitz, Germany); diisopropylethylamine, DMF and 4-methylpiperidine were from Sigma-Aldrich; TFA (BioGrade) was from Halocarbon (Peachtree Corners, GA). ^{15}N-phenylalanine, ^{15}N-valine, ^{15}N-glycine, ^{15}N-leucine and ^{15}N-isoleucine were purchased from Cambridge Isotope Laboratories (Tewksbury, MA) and Fmoc-protected in-house. All other chemicals were purchased from Sigma-Aldrich.

Peptide synthesis: automated microwave peptide synthesis was performed on a Liberty Blue (CEM) synthesizer. Typically, syntheses were performed on a 0.1 mmol scale by Fmoc/tBu solid-phase peptide synthesis (Fmoc-SPPS; side-chain protection: Asn(Trt), Asp(OtBu), Cys(Trt), Glu(OtBu), His(Trt), Lys(Boc), Ser(tBu), Tyr(tBu)). Peptide-$^{\alpha}$carboxylates were synthesized on a preloaded Fmoc-Lys(Boc)-Wang-PS-resin (loading: 0.68 mmol/g, mesh size: 180-333). Peptide-$^{\alpha}$hydrazides were prepared on a 2-CT-$NHNH_2$ resin prepared in-house (loading: 0.4 mmol/g, mesh size: 200-400). The coupling reactions were performed by adding amino acids dissolved in DMF (2.5 mL, 0.2 M), the coupling reagent DIC in DMF (1.0 mL, 0.5 M) and Oxyma Pure in DMF (0.5 mL, 1 M) to the respective resin. Standard couplings were performed at 90 °C for 2 min. Standard deprotections were performed using 20% (*v/v*) 4-methylpiperidine in DMF at 90 °C for 1 min.

Prior to cleavage, peptidyl resin was washed with DCM and dried under vacuum. Peptides were cleaved and fully deprotected by treatment with TFA/DTT/TIPS/H_2O (92.5:2.5:2.5:2.5, *v/w/v/v*) for 2 h (1 mL of cleavage reagent for 100 mg of resin). The reaction mixtures were diluted 20-fold with diethyl ether (-20°C) for precipitation of the crude peptides. After centrifugation, the precipitates were dissolved in H_2O/CH_3CN (1:1, *v/v*) containing TFA (0.1%, *v/v*) and lyophilized.

Analytical HPLC: Analytical reversed-phase HPLC was performed on a Shimadzu Nexera XR UHPLC instrument. Alternatively, a Thermo Scientific Dionex 3000 was used. A Phenomenex Kinetex XB-C18 column (50×2.1 mm, 100 Å, 2.6 µm) was used at 1 mL/min with a gradient of water with TFA (0.1%, *v/v*) and acetonitrile with TFA (0.08%, *v/v*).

Preparative HPLC: Purifications of peptides were performed on a preparative Shimadzu HPLC instrument equipped with two LC-20AP pumps and an SPD-20A Prominence UV/vis detector connected to an FRC-10A fraction collector. Phenomenex Kinetex XB-C18 column (250×21.2 mm, 100 Å, 5 µm) was used at 10 mL/min with a gradient of water with TFA (0.1%, *v/v*) and acetonitrile with TFA (0.08%, *v/v*).

LC-MS: Peptide masses were measured on a LC/MS instrument equipped with a Thermo Scientific Accela UHPLC (Hypers II GOLD column, 50×2.1 mm, 1.9 µm) integrated with a Thermo Scientific LCQ Fleet ion-trap. Deconvolution of data was performed in MagTran 1.03 (Amgen, Thousand Oaks, CA). More precise MS measurements for larger protein products have been performed using a Thermo Scientific Q Exactive hybrid quadrupole-Orbitrap mass-spectrometer. Direct infusion into electrospray ionization source was performed from solutions of proteins in H_2O/CH_3CN (1:1, *v/v*) containing formic acid (0.1%, *v/v*).

Size-exclusion chromatography: SEC was performed on a Shimadzu Nexera XR UHPLC instrument. A Phenomenex Yarra 3µm SEC 2000 column was used (300×4.6 mm, 2.8 µm) with an isocratic mobile phase composed of NaH_2PO_4 (10 mM), Na_2HPO_4 (40 mM), NaCl (100 mM) and NaN_3 (0.05%, *w/v*), pH 7.5, at a flow of 0.5 mL/min, at 27 °C, detected at 280 nm. A gel filtration markers kit for protein molecular weights (6,500-66,000 Da) was used for calibration. The kit was composed of Blue Dextran (for the determination of the void volume), aprotinin from bovine lung (6.5 kDa), cytochrome *c* from equine heart (12.4 kDa), carbonic anhydrase from bovine erythrocytes (29 kDa) and albumin from bovine serum (66 kDa) and was purchased from Sigma-Aldrich.

NMR spectroscopy: 1D ^{1}H spectra shown in Figure S7 were recorded on a Bruker Avance III 600 MHz spectrometer. The spectra and data depicted in Figure 4c, Figures S8-S14 were recorded on a Bruker Avance III 700 MHz spectrometer equipped with a TCI cryoprobe. For 2D, relaxation and DOSY spectra acquisitions, a sample of **TofT-3** in a 3 mm tube was prepared at concentration of 200 µM (volume 180 µL) in 50 mM phosphate buffer at pH 7 (90:10% *v/v* H_2O/D_2O). The ^{1}H NOESY spectrum (2048 x 600 points) was recorded at 35 °C with a mixing time of 150 ms. 32 scans were acquired for each t1 increment leading to a total experimental time of 12 hours. The water signal was suppressed using a Watergate sequence. The ^{1}H-^{13}C HSQC spectrum (2048 x 400 points) was recorded at 35 °C with 32 scans per t1 increment. The carbon bandwidth and carrier frequency were set to 70 ppm and 40 ppm, respectively and the quadrature detection was obtained using pulse field gradients selection of echo and anti-echo coherences. The experimental acquisition time was 5 hours. The ^{1}H-^{15}N HSQC spectrum (2048 x 128 points) was recorded at 35 °C with 200 scans per t1 increment (6 hours experimental time). The nitrogen bandwidth and carrier frequency were set to 30 ppm and 116.5 ppm, respectively and the quadrature detection was obtained using a STATES-TPPI phase program. The ^{15}N relaxation rates were measured from HSQC type experiments as described.(1) For ^{15}N T_1 relaxation, intensities were extracted from a set of 9 spectra recorded with relaxation delays ranging between 20 ms and 1 s. For ^{15}N T_2 relaxation, intensities were extracted from a set of 10 spectra recorded with relaxation delays ranging from 0 to 130 ms, with ^{15}N 180° pulses applied every 0.9 ms at a field strength of

4.2 kHz. The 2D spectra were processed using nmrPipe program[(2)] and the intensities were measured using CcpNmr.[(3)] The relaxation rates were computed from non-linear fits of the time dependent intensities by either a single or double exponential decay function using in-house Python scripts. The values of the T_1 and T_2 relaxation times provide an estimate of the global correlation time (τ_c) of **TofT-3** using the approximation provided by Farrow et al:[(1)]

$$\boldsymbol{\tau_c} = \sqrt{\frac{6\frac{T1}{T2}-7}{4\omega_N^2}},$$

where T_1 = spin-lattice relaxation time constant, T_2 = spin-spin relaxation time constant and ω_N = ^{15}N Larmor frequency.

This gives a value between 9.8 and 13.2 ns, in agreement with the expected correlation time of a protein of ~200 amino-acids at 35 °C (11.5 ns as estimated from the model of Daragan).[(4)]

DOSY spectra were recorded for **TofT-3** as a function of temperature to get an estimate of the hydrodynamic radius using TRIS as an internal reference.[(5)] The DOSY spectrum was performed using the led_es sequence at 35 °C. The main diffusion time (Δ) was set to 200 ms. The diffusion pulse length was 1.8 ms.

Fluorescence spectroscopy: Fluorescence was recorded on a Victor X5 2030-0050 multi-label HTS plate-reader (PerkinElmer) with Nunc black standard 96-well plates sealed with transparent ThermalSeal RT film (Sigma-Aldrich). The excitation wavelength was 485 nm (ThT, 4-(3,6-dimethyl-1,3-benzothiazol-3-ium-2-yl)-N,N-dimethylaniline), 405 nm (bis-ANS, 4,4'-dianilino-1,1'-binaphthyl-5,5'-disulfonic acid), or 531 nm (Nile Red, 9-diethylamino-5-benzo[a]phenoxazinone). The emitted fluorescence was collected at 535 nm (ThT), 460 nm (bis-ANS), and 595 nm (Nile Red). Data are averages of three measurements. Concentrations of **TofT-1** and **TofT-2** were 10 µM and the concentration of dye (ThT, bis-ANS or Nile Red) was 30 µM in sodium phosphate buffer (100 µL, 50 mM, pH 7.5) with NaCl (100 mM) and NaN_3 (0.05%, *w/v*).

Kinetics of amyloid growth: Continuous amyloid growth assays were performed on a Victor X5 2030-0050 multi-label HTS plate-reader (PerkinElmer) with Nunc black standard 96-well plates sealed with transparent ThermalSeal RT film (Sigma-Aldrich). Fluorescence readings were taken at 30 °C, with shaking for 1 s every 10 min; reactions were monitored for up to five days. Excitation/emission wavelengths were set with optical filters. Samples had a volume of 100 µL of a sodium phosphate buffer (50 mM, pH 7.5) with NaCl (100 mM) and NaN_3 (0.05%, *w/v*) containing 33.3 µM of "covalent trimer" [20-41]β2m, 100 µM of ThT and 11.1 µM of *N*-methylated peptide inhibitors. "Covalent trimer" [20-41]β2m was dissolved

in DMSO as a 3.33 mM stock solution and was added to the phosphate buffer by 100-fold dilution. The *N*-methylated peptide inhibitors were directly dissolved in the buffer. The final concentration of DMSO in each sample was 1% (*v/v*).

Transmission electron microscopy: Carbon Type B (15-25 nm) formvar-coated Cu electron microscopy grids (400 mesh; Ted Pella, Redding, CA) were placed coated-side-down for 60 s on the amyloid sample. The grids were washed with deionized water, stained with uranyl acetate (2%, *w/v*) for 20 s, and then air-dried. Images were taken on a Philips CM12 electron microscope (80 kV).

Molecular modeling: Molecular graphics and analyses were performed using MOE software.[(6)] Model 1 from PDB ID: 2E8D (solid-state NMR structure of [20-41]β2m amyloid protofilament) was selected. A stack of three β-arch-containing trimers composed of nine copies of [20-41]β2m was built along the fiber axis. Central linkers were attached to each of the three trimers. Energies of the models were minimized by using the minimization force field AMBER10:EHT, with R-Field as a solvation mode. Minimizations were done stepwise: first, backbones were fixed and models were minimized, thus allowing side-chain and central linker reorganization; and a second minimization afforded reorganization of peptide backbone and side chains simultaneously. *C-N* linkers 1 and 2 were then introduced and the models were minimized using the same protocol as before. Finally, *N*-methylations were inserted by replacing the hydrogen atom of the amide bonds by a methyl group and models were minimized.

Chemical synthesis

Preparation of the hydrazide (2-CT-NHNH$_2$) resin:[(7)] The 2-CT-OH resin (3 g, 1.6 mmol/g active groups, mesh size 200-400) was placed in a three-necked round bottom flask. It was flushed several times with argon. Dry dichloromethane (28 mL) was added. The mixture was gently stirred allowing the resin to swell. Thionyl chloride (450 µL, 6.2 mmol, 1.3 equiv) was slowly added at 0°C. The suspension was stirred under argon for 2 h allowing it to slowly warm up to room temperature. The solvent was removed and the resin was washed with DCM and DMF. The 2-CT-Cl resin was then swollen for 20 min in DMF (18 mL). A mixture of hydrazine monohydrate (700 µL, 14.4 mmol, 3 equiv) and DIEA (2 mL, 11.5 mmol, 2.4 equiv) in DMF (4 mL) was added to the resin at 0°C. The suspension was stirred at r.t. for 90 min. The reaction was quenched by the addition of methanol (300 µL). The resin was washed with DMF, water, DMF, methanol and diethyl ether and dried under reduced pressure. Final mass of the resin: 3 g.

Synthesis of the central linker: 3,5-bis(((allyloxy)carbonyl)amino)benzoic acid

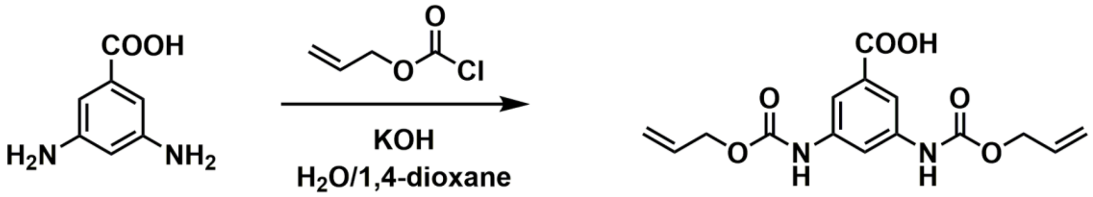

Potassium hydroxide (1.09 g, 19.5 mmol, 3 equiv) was added to a mixture of 3,5-diaminobenzoic acid (1.05 g, 6.5 mmol, 1 equiv) in water (40 mL). It was stirred until a clear solution was obtained. 1,4-dioxane (30 mL) was added to the reaction mixture followed by a dropwise addition of a solution of allyl chloroformate (1.52 mL, 14.3 mmol, 2.2 equiv) in 1,4-dioxane (10 mL). It was stirred at r.t. overnight. The volatiles were removed under reduced pressure. Water (100 mL) was added to the resulting paste and the pH was adjusted to 8-9 with sat. Na_2CO_3(aq). The aqueous solution was washed with ethyl acetate (2 x 50 mL). The aqueous layer was then treated with 6 M HCl(aq) to lower the pH to 3 and extracted with ethyl acetate (3 x 50 mL). The organic layers were gathered, washed with brine, dried over anhydrous sodium sulfate and concentrated under reduced pressure. The isolated yield was 2.0 g (95%). LC-MS (ESI): Average isotope calculated 320.3 Da [*M*]; found: 320.8 Da. Elemental analysis calculated for $C_{15}H_{16}N_2O_6$: C, 56.25; H, 5.04; N, 8.75; found: C, 55.69; H, 5.11; N 8.70.

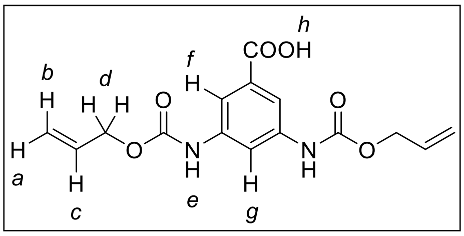

^{1}H NMR (400 MHz, DMSO-d_6) δ, ppm: 4.61 (4H, ddd, H_d, $^3J_{dc}$ = 5.4 Hz, $^4J_{ad}$ = 1.5 Hz, $^4J_{bd}$ = 1.5 Hz), 5.24 (2H, ddt, H_a, $^2J_{ab}$ = 1.5 Hz, $^3J_{ac}$ = 10.5 Hz, $^4J_{ad}$ = 1.5 Hz), 5.36 (2H, ddt, H_b, $^2J_{ab}$ = 1.5 Hz, $^3J_{bc}$ = 17.2 Hz, $^4J_{bd}$ = 1.5 Hz), 5.98 (2H, ddt, H_c, $^3J_{bc}$ = 17.2 Hz, $^3J_{ac}$ = 10.7 Hz, $^3J_{dc}$ = 5.4 Hz), 7.73 (2H, d, H_f, $^4J_{fg}$ = 2.0 Hz), 7.94 (1H, t, H_g, $^4J_{fg}$ = 2.0 Hz), 9.91 (2H, s, H_e), 12.92 (1H, s, H_h).

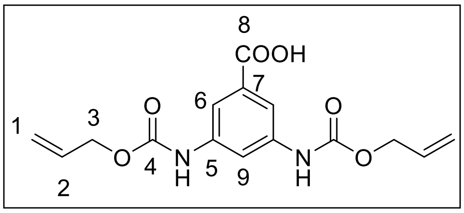

^{13}C NMR (101 MHz, DMSO-d_6) δ, ppm: 65.20 (C3), 112.72 (C9), 113.94 (C6), 118.06 (C1), 132.15 (C7), 133.71 (C2), 140.20 (C5), 153.63 (C4), 167.54 (C8). The assignment of the carbon signals to the appropriate proton resonances is based on DEPT, HSQC and HMBC measurements.

Procedures for the on-resin functionalization of the ornithine residue (0.1 mmol scale):

*Boc protection of the last coupled residue (<u>This step is only required for the synthesis of</u> <u>**peptides B** and **H** (see Tables S1 and S2)</u>)*: After chain assembly and deprotection of the last Fmoc-group, the resin was swollen in DMF. *N*-terminal Boc protection of *N*-terminal serine was performed with the addition of Boc-anhydride (87.5 mg, 0.4 mmol, 4 equiv, in 1 mL of DMF) and DIEA (68 µL, 0.4 mmol, 4 equiv). It was let to react at r.t. for 30 min with manual stirring. Kaiser test was negative.

<u>*Procedures below were employed for the synthesis of* **peptides B, D, F, H, J** *and* **K** *(see Tables S1-S3):*</u>

Alloc deprotection of ornithine: The resin was swollen in DCM purged with argon. A solution of phenylsilane (296 µL, 2.4 mmol, 24 equiv) in DCM (1 mL) purged with argon was added to the resin. Alloc deprotection was carried out upon the addition of a solution of $Pd(PPh_3)_4$ (58 mg, 0.05 mmol, 0.5 equiv) in DCM (2 mL) purged with argon. It was let to react at r.t. for 30 min with manual stirring. Kaiser test was positive.

Linker coupling to ornithine side chain: The resin was swollen in DMF. A solution of 3,5-bis(((allyloxy)carbonyl)amino)benzoic acid (128 mg, 0.4 mmol, 4 equiv), HATU (144 mg, 0.38 mmol, 3.8 equiv) and DIEA (100 µL, 0.6 mmol, 6 equiv) in 1 mL of DMF was prepared (2 min of pre-activation) and added to the resin. It was let to react at r.t. for 30 min. Kaiser test was negative.

Alloc deprotection of the linker: Same procedure as before (repeated twice). Chloranil test was positive.

Bromoacetylation of the linker: The resin was swollen in DMF. A solution of bromoacetic acid (167 mg, 1.2 mmol, 12 equiv) and *N*,*N*'-diisopropylcarbodiimide (86 µL, 1.1 mmol, 11.2 equiv) in 2 mL of DMF was prepared (2 min of pre-activation) and added to the resin. It was let to react at r.t. for 60 min. Chloranil test was negative.

***Procedure for the synthesis of* NMT-A-1:**

Peptide **A** (30.1 mg, 12 µmol) and peptide **B** (17.5 mg, 6 µmol) (see Table S1) were dissolved in 5 mL of buffer ([Na_2HPO_4] = 0.1 M, [Gn·HCl] = 6 M, [Na_4·EDTA] = 5 mM, pH = 8.0). The pH was adjusted to 8.0 with a 1 M $Na_2CO_{3(aq)}$ solution. It was let to react at 30°C for 2 h. The pH was then lowered to 3 with 6 M HCl(aq). Sodium nitrite, solubilized in argon purged pH 3 buffer (30 µmol, c = 0.2 M, 150 µL, 5 equiv), was added to the reaction mixture. It was let to react at -10°C for 15 min under argon. Sodium 2-mercaptoethanesulfonate, solubilized in argon purged pH 7 buffer (300 µmol, c = 0.2 M, 1.5 mL, 50 equiv) was added.

It was let to react at -10°C for 2 h under argon. The reaction mixture was diluted and directly injected into preparative HPLC and the product was purified using a Phenomenex Kinetex XB-C18 (250 x 21.2 mm, 100 Å, 5 µm) column with a gradient of water with TFA (0.1%, *v/v*) and acetonitrile with TFA (0.08%, *v/v*). Pure fractions of product were combined and lyophilized to give a white solid. *This procedure was also employed for the synthesis of* **NMT-A-2** *(see Table S2).*

***Procedure for the synthesis of* CT-1:**

Peptide **C** (34.8 mg, 13.9 µmol) and peptide **D** (22.0 mg, 6.9 µmol) (see Table S1) were dissolved in 9.2 mL of buffer ([Na_2HPO_4] = 0.1 M, [Gn·HCl] = 6 M, [Na_4·EDTA] = 5 mM, pH = 8.0). The pH was adjusted to 8.0 with a 1 M $Na_2CO_{3(aq)}$ solution. It was let to react at 30°C for 2 h. Methoxyammonium chloride (154 mg, 16.7 mg/mL) was added to the reaction mixture. The pH was then lowered to 4 with 6 M HCl(aq). It was let to react at 30°C for 2 h. The reaction mixture was diluted and directly injected into preparative HPLC and the product was purified using a Phenomenex Kinetex XB-C18 (250 x 21.2 mm, 100 Å, 5 µm) column with a gradient of water with TFA (0.1%, *v/v*) and acetonitrile with TFA (0.08%, *v/v*). Pure fractions were combined and lyophilized to give a white solid. *This procedure was also employed for the synthesis of* **NMT-B-1**, **NMT-B-2** *and* **CT-2** *(see Tables S1-S3).*

First ligation step in the synthesis of* TofT-2 *construct:

NMT-A-2 (10.0 mg, 1.3 µmol) and **CT-1** (10.1 mg, 1.3 µmol) were dissolved in 1.0 mL of argon purged buffer 1 ([Na_2HPO_4] = 0.2 M, [Gn·HCl] = 6 M, pH = 7.0) containing MPAA (13 mM, 12.6 µmol, 10 equiv) and TCEP (20 mM, 20.2 µmol, 16 equiv). The pH was adjusted to 7.0 with a 1 M $Na_2CO_{3(aq)}$ solution after peptide dissolution. It was let to react at 40°C for 2 h under argon. The reaction mixture was diluted with 2 mL of buffer 1. Bromoacetamide (158 µL, 0.2 M, 32.5 µmol, 25 equiv) was solubilized in buffer 1 and added to the reaction mixture. It was let to react at 40°C for 30 min. The pH was adjusted to 3.0 with a 6 M $HCl_{(aq)}$ solution. At -10°C, sodium nitrite (64 µL, 0.2 M, 12.6 µmol, 10 equiv) was solubilized in buffer 2 ([Na_2HPO_4] = 0.2 M, [Gn·HCl] = 6 M, pH = 3.0) and was added to the reaction mixture. It was let to react for 20 min. At -10°C, sodium 2-mercaptoethanesulfonate (640 µL, 0.2 M, 126 µmol, 100 equiv) was solubilized in buffer 1 and added to the reaction mixture. It was let to react for 2 h. The reaction mixture was diluted and directly injected into preparative HPLC. The product was purified using a Phenomenex Kinetex XB-C18 (250 x 21.2 mm, 100 Å, 5 µm) column with a gradient of water with TFA (0.1%, *v/v*) and acetonitrile with TFA (0.08%, *v/v*). Pure fractions were combined and lyophilized to give a white solid of the ligation product (**DofT-2** = "dimer of trimers"-2). Isolated yield: 7.0 mg (34 %). *This procedure was also employed for the synthesis of* **DofT-1** *(product of ligation of* **NMT-A-1** *and* **CT-1**, *isolated*

yield: 4.2 mg (54 %)) and **DofT-3** *(product of ligation of* **NMT-A-2** *and* **CT-2**, *isolated yield: 5.0 mg (29 %)).*

DofT-1: LC-MS (ESI): [*M* + 8H$^+$] = 1990.8 *m/z*; [*M* + 9H$^+$] = 1769.6 *m/z*; [*M* + 10H$^+$] = 1592.6 *m/z*; [*M* + 11H$^+$] = 1448.1 *m/z*; [*M* + 12H$^+$] = 1327.6 *m/z*; [*M* + 13H$^+$] = 1225.4 *m/z*; [*M* + 14H$^+$] = 1138.1 *m/z*; [*M* + 15H$^+$] = 1062.4 *m/z*; [*M* + 16H$^+$] = 996.3 *m/z*; $C_{720}H_{1061}N_{173}O_{222}S_7$; Average isotope calculated 15916.8 Da [*M*]; found: 15919.2 Da. Here, there is a discrepancy between the calculated and experimental mass due to an incorrect calibration of the LC-MS apparatus. However, the final product **TofT-1** (after ligation of **DofT-1** and **NMT-B-1**) has correct mass (see Figure S4).

DofT-2: LC-MS (ESI): [*M* + 9H$^+$] = 1773.9 *m/z*; [*M* + 10H$^+$] = 1596.6 *m/z*; [*M* + 11H$^+$] = 1451.6 *m/z*; [*M* + 12H$^+$] = 1330.8 *m/z*; [*M* + 13H$^+$] = 1228.5 *m/z*; [*M* + 14H$^+$] = 1140.8 *m/z*; [*M* + 15H$^+$] = 1064.9 *m/z*; [*M* + 16H$^+$] = 998.3 *m/z*; $C_{723}H_{1067}N_{173}O_{222}S_7$; Average isotope calculated 15958.90 Da [*M*]; found: 15957.0 Da.

DofT-3: LC-MS (ESI): [*M* + 8H$^+$] = 1996.5 *m/z*; [*M* + 9H$^+$] = 1774.5 *m/z*; [*M* + 10H$^+$] = 1597.4 *m/z*; [*M* + 11H$^+$] = 1452.1 *m/z*; [*M* + 12H$^+$] = 1331.3 *m/z*; [*M* + 13H$^+$] = 1229.0 *m/z*; [*M* + 14H$^+$] = 1141.2 *m/z*; [*M* + 15H$^+$] = 1065.2 *m/z*; [*M* + 16H$^+$] = 998.7 *m/z*; $C_{723}H_{1067}N_{173}O_{222}S_7$; Average isotope calculated 15964.0 Da [*M*]; found: 15963.5 Da.

***Second ligation step in the synthesis of* TofT-2:**

NMT-B-2 (6.5 mg, 0.8 µmol) and **DofT-2** (6.6 mg, 0.4 µmol) were dissolved in 200 µL of argon purged buffer 1 ([Na_2HPO_4] = 0.2 M, [Gn·HCl] = 6 M, pH = 7.0) containing MPAA (11 mM, 2 µmol, 5 equiv) and TCEP (34 mM, 6.6 µmol, 16 equiv). The pH was adjusted to 6.8 with a 1 M $Na_2CO_{3(aq)}$ solution after peptide dissolution. It was let to react at 40°C for 30 min under argon. The reaction mixture was diluted with 400 µL of buffer 1. Bromoacetamide (51 µL, 0.2 M, 13.3 µmol, 25 equiv) was solubilized in buffer 1 and added to the reaction mixture. It was let to react at r.t. for 30 min. The reaction mixture was diluted and directly injected into preparative HPLC using a Phenomenex Kinetex XB-C18 (250 x 21.2 mm, 100 Å, 5 µm) column with a gradient of water with TFA (0.1%, *v/v*) and acetonitrile with TFA (0.08%, *v/v*). Pure fractions were combined and lyophilized to give a white solid. Isolated yield: 3.7 mg (38 %). *This procedure was also employed for the synthesis of* **TofT-1** *(isolated yield: 3.2 mg (25 %)) and* **TofT-3** *(isolated yield: 2.1 mg (40 %).*

TofT-1: ESI-MS-Orbitrap: [*M* + 12H$^+$] = 1989.9 *m/z*; [*M* + 13H$^+$] = 1836.9 *m/z*; [*M* + 14H$^+$] = 1705.8 *m/z*; [*M* + 15H$^+$] = 1592.0 *m/z*; [*M* + 16H$^+$] = 1492.7 *m/z*; [*M* + 17H$^+$] = 1404.9 *m/z*; [*M* + 18H$^+$] = 1326.9 *m/z*; [*M* + 19H$^+$] = 1257.1 *m/z*; [*M* + 20H$^+$] = 1194.3 *m/z*; [*M* + 21H$^+$] =

1137.6 *m/z*; $C_{1084}H_{1596}N_{262}O_{332}S_8$; Average isotope calculated 23866.7 Da [*M*]; found: 23866.4 Da.

TofT-2: ESI-MS-Orbitrap: [*M* + 12H$^+$] = 1996.9 *m/z*; [*M* + 13H$^+$] = 1843.3 *m/z*; [*M* + 14H$^+$] = 1711.8 *m/z*; [*M* + 15H$^+$] = 1597.6 *m/z*; [*M* + 16H$^+$] = 1497.9 *m/z*; [*M* + 17H$^+$] = 1409.9 *m/z*; [*M* + 18H$^+$] = 1331.6 *m/z*; [*M* + 19H$^+$] = 1261.6 *m/z*; [*M* + 20H$^+$] = 1198.5 *m/z*; [*M* + 21H$^+$] = 1141.5 *m/z*; $C_{1090}H_{1608}N_{262}O_{332}S_8$; Average isotope calculated 23950.8 Da [*M*]; found: 23950.6 Da.

TofT-3: ESI-MS-Orbitrap: [*M* + 12H$^+$] = 1997.1 *m/z*; [*M* + 13H$^+$] = 1843.8 *m/z*; [*M* + 14H$^+$] = 1712.1 *m/z*; [*M* + 15H$^+$] = 1597.9 *m/z*; [*M* + 16H$^+$] = 1498.2 *m/z*; [*M* + 17H$^+$] = 1410.2 *m/z*; [*M* + 18H$^+$] = 1331.9 *m/z*; [*M* + 19H$^+$] = 1261.8 *m/z*; [*M* + 20H$^+$] = 1198.8 *m/z*; [*M* + 21H$^+$] = 1141.7 *m/z*; $C_{1090}H_{1608}N_{262}O_{332}S_8$; Average isotope calculated 23955.9 Da [*M*]; found: 23955.5 Da.

Synthesis of covalently tethered N-methylated inhibitors:

Covalent dimer [20-41]β2m with N-Me-Asn5, N-Me-Val18: **Peptide G** (Table S2, 10.0 mg, 4.0 µmol) were dissolved in 5 mL of buffer ([Na_2HPO_4] = 0.1 M, [Gn·HCl] = 6 M, [EDTA.Na_4] = 5 mM, pH = 7.0). The pH was adjusted to 9.0 with 6 M $NaOH_{(aq)}$ after peptide dissolution. It was let to react at 30°C for 4 days. The crude peptide was purified by preparative HPLC using a Phenomenex Kinetex XB-C18 (100 x 21.2 mm, 100 Å, 5 µm) column with a gradient of water with TFA (0.1%, *v/v*) and acetonitrile with TFA (0.08%, *v/v*). Pure fractions were combined and lyophilized to give a white solid. The isolated yield was 3.2 mg (31%). LC-MS (ESI): [*M* + 3H$^+$] = 1684.4 *m/z*; [*M* + 4H$^+$] = 1263.7 *m/z*; [*M* + 5H$^+$] = 1011.1 *m/z*; [*M* + 6H$^+$] = 842.5 *m/z*; $C_{230}H_{344}N_{54}O_{70}S_2$; Average isotope calculated 5149.7 Da [*M*]; found: 5050.8 Da.

Covalent dimer [20-41]β2m with N-Me-Leu4, N-Me-Asp19: **Peptide I** (Table S2, 10.0 mg, 4.0 µmol) were dissolved in 5 mL of buffer ([Na_2HPO_4] = 0.1 M, [Gn·HCl] = 6 M, [EDTA.Na_4] = 5 mM, pH = 7.0). The pH was adjusted to 9.0 with 6 M $NaOH_{(aq)}$ after peptide dissolution. It was let to react at 30°C for 4 days. The crude peptide was purified by preparative HPLC using a Phenomenex Kinetex XB-C18 (100 x 21.2 mm, 100 Å, 5 µm) column with a gradient of water with TFA (0.1%, *v/v*) and acetonitrile with TFA (0.08%, *v/v*). Pure fractions were combined and lyophilized to give a white solid. The isolated yield was 4.0 mg (39%). LC-MS (ESI): [*M* + 3H$^+$] = 1684.0 *m/z*; [*M* + 4H$^+$] = 1263.5 *m/z*; [*M* + 5H$^+$] = 1011.3 *m/z*; [*M* + 6H$^+$] = 842.5 *m/z*; $C_{230}H_{344}N_{54}O_{70}S_2$; Average isotope calculated 5149.7 Da [*M*]; found: 5049.8 Da.

Covalent trimer [20-41]β2m with N-Me-Asn5, N-Me-Val18: **Peptide G** (Table S2, 8.5 mg, 6 µmol) was suspended in NH_4HCO_3 (3.4 mL, 200 mM, pH 8) containing EDTA (5 mM). Then, *N,N′,N″*-(benzene-1,3,5-triyl)-tris(2-bromoacetamide) (840 µL, 1 mM in acetonitrile, 1.5 µmol) was added. The suspension was vortexed and sonicated until a clear solution was obtained. The reaction mixture was shaken on a ThermoMixer (600 rpm) at 30°C for 2 h and then lyophilized. The crude solid was dissolved in guanidine hydrochloride (Gn·HCl, 6 M, 1 mL per 10 mg of crude solid) and immediately purified by preparative HPLC using a Phenomenex Kinetex XB-C18 (100 x 21.2 mm, 100 Å, 5 µm) column with a gradient of water with TFA (0.1%, *v/v*) and acetonitrile with TFA (0.08%, *v/v*). Pure fractions were combined and lyophilized to give a white solid. The isolated yield based on the linker was 3.6 mg (55%). LC-MS (ESI): $[M + 4H^+]$ = 1956.2 *m*/*z*; $[M + 5H^+]$ = 1565.3 *m*/*z*; $[M + 6H^+]$ = 1304.2 *m*/*z*; $[M + 7H^+]$ = 1118.2 *m*/*z*; $[M + 8H^+]$ = 978.6 *m*/*z*; $[M + 9H^+]$ = 869.8 *m*/*z*; $C_{357}H_{528}N_{84}O_{108}S_3$; Average isotope calculated 7820.8 Da [*M*]; found: 7820.1 Da.

Covalent trimer [20-41]β2m with N-Me-Leu4, N-Me-Asp19: **Peptide I** (Table S2, 8.5 mg, 3.4 µmol) was suspended in NH_4HCO_3 (3.4 mL, 200 mM, pH 8) containing EDTA (5 mM). Then, *N,N′,N″*-(benzene-1,3,5-triyl)-tris(2-bromoacetamide) (840 µL, 1 mM in acetonitrile, 1.5 µmol) was added. The suspension was vortexed and sonicated until a clear solution was obtained. The reaction mixture was shaken on a ThermoMixer (600 rpm) at 30°C for 2 h and then lyophilized. The crude solid was dissolved in guanidine hydrochloride (Gn·HCl, 6 M, 1 mL per 10 mg of crude solid) and immediately purified by preparative HPLC using a Phenomenex Kinetex XB-C18 (100 x 21.2 mm, 100 Å, 5 µm) column with a gradient of water with TFA (0.1%, *v/v*) and acetonitrile with TFA (0.08%, *v/v*). Pure fractions were combined and lyophilized to give a white solid. The isolated yield based on the linker was 4.8 mg (73%). LC-MS (ESI): $[M + 4H^+]$ = 1956.4 *m*/*z*; $[M + 5H^+]$ = 1564.9 *m*/*z*; $[M + 6H^+]$ = 1304.4 *m*/*z*; $[M + 7H^+]$ = 1118.2 *m*/*z*; $[M + 8H^+]$ = 978.6 *m*/*z*; $[M + 9H^+]$ = 869.8 *m*/*z*; $C_{357}H_{528}N_{84}O_{108}S_3$; Average isotope calculated 7820.8 Da [*M*]; found: 7820.5 Da.

Table S1. Building blocks used to assemble **TofT-1** construct

	Building block	***Analytical characterization***
Covalent trimers:		
**NMT-A-1**	*N*-methylated amino acids: ***N*-Me-Ile16** 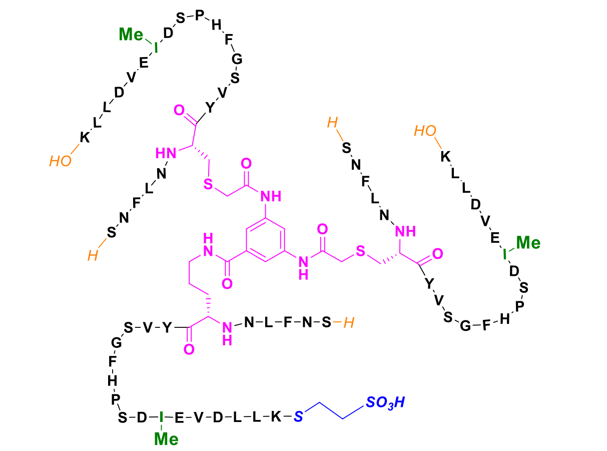	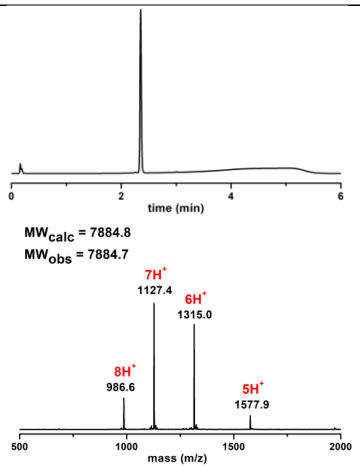 The isolated yield: 32.4 mg (68%).
**CT-1**	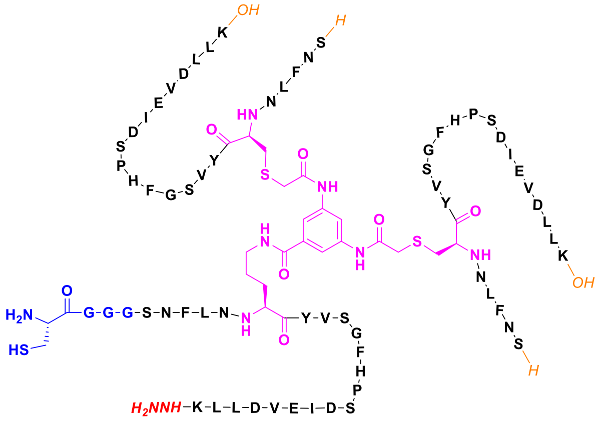	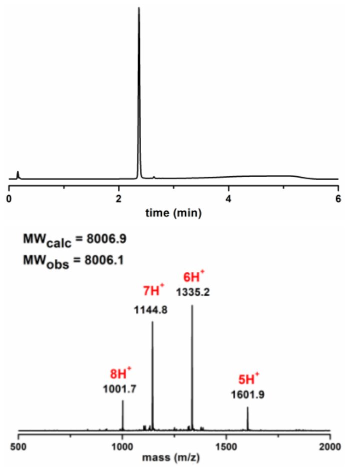 The isolated yield: 22.5 mg (41%).
**NMT-B-1**	*N*-methylated amino acids: ***N*-Me-Glu17** 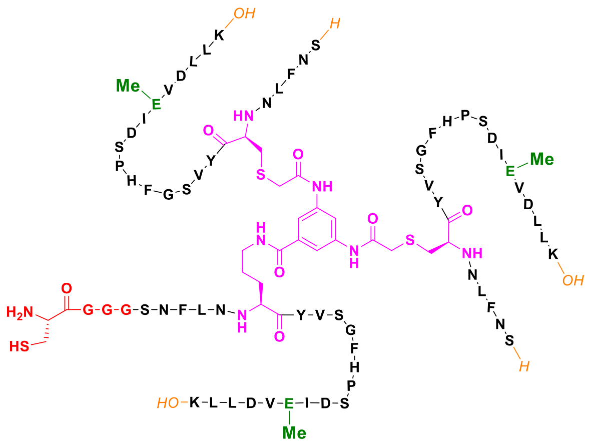	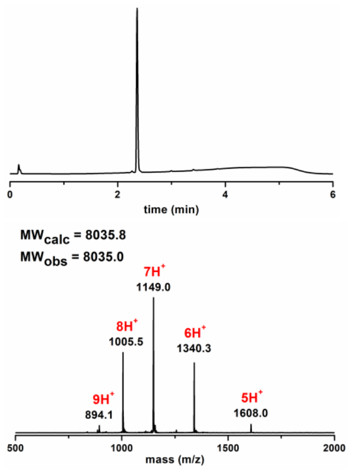 The isolated yield: 20.3 mg (54%).

Continued on p. 13

Continued from p. 12

Peptides used in the synthesis of covalent trimers:

peptide A:

N-methylated amino acids: ***N*-Me-Ile16**

<u>*sequence:*</u> SNFLN**Cys**YVSGFHPSD***N*-Me-Ile**EVDLLK

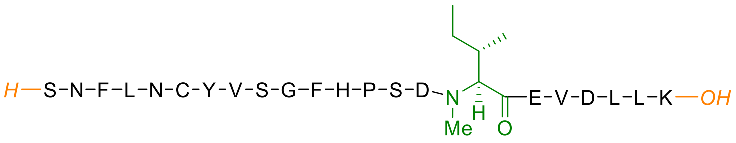

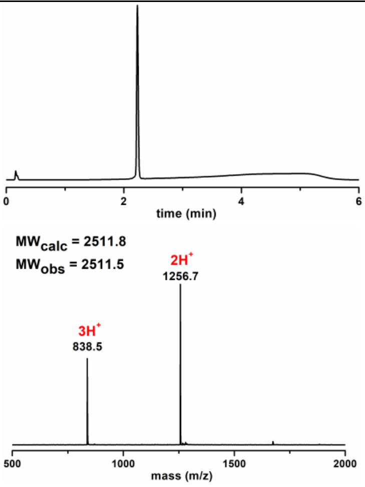

The isolated yield: 97 mg (37% based on the resin loading).

peptide B:

N-methylated amino acids: ***N*-Me-Ile16**

<u>*sequence:*</u>

SNFLN***Orn(linker)***YVSGFHPSD***N*-Me-Ile**EVDLLK-***NHNH*$_2$**

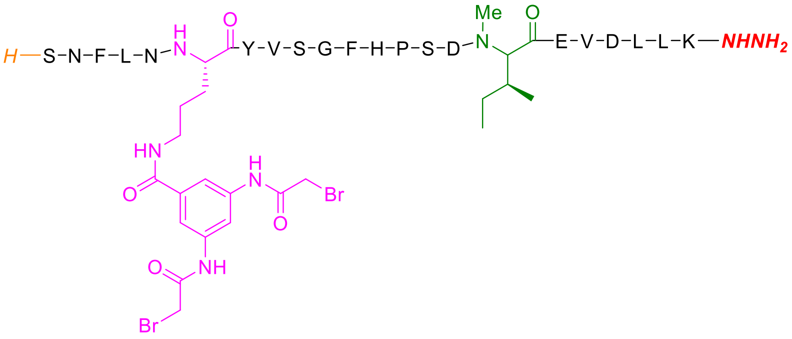

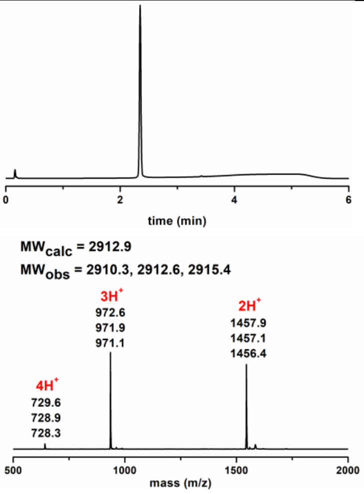

The isolated yield: 84 mg (29% based on the resin loading).

peptide C: [20-41]β2m-fragment

<u>*sequence:*</u> *H-*SNFLN**Cys**YVSGFHPSDIEVDLLK*-OH*

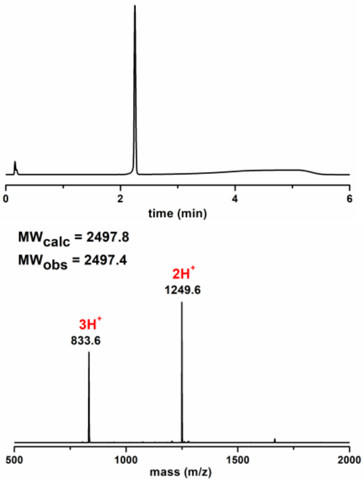

The isolated yield: 104 mg (42% based on the resin loading).

Continued on p. 14

Continued from p. 13

peptide D:

sequence:

Thz-GGG-SNFLN***Orn(linker)***YVSGFHPSDIEVDLLK-***NHNH$_2$***

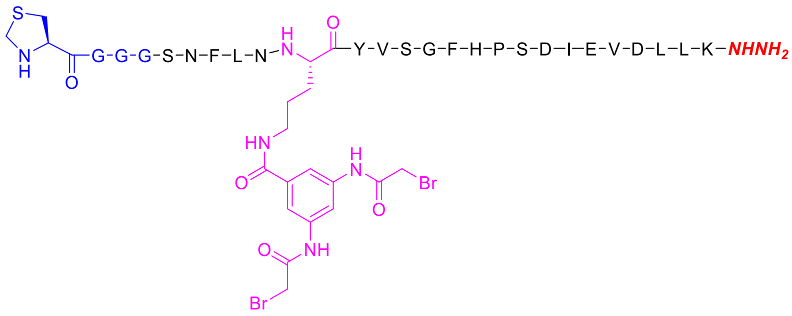

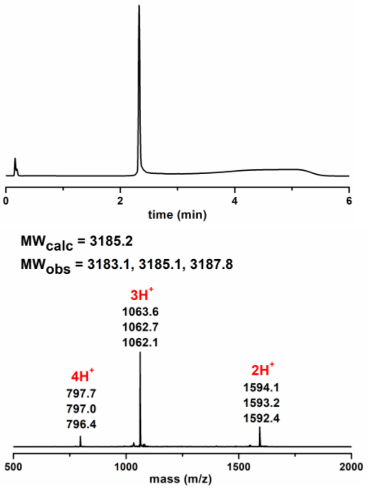

The isolated yield: 140 mg (44% based on the resin loading).

peptide E:

N-methylated amino acids: ***N*-Me-Glu17**

sequence:

H-SNFLN**Cys**YVSGFHPSDI***N*-Me-Glu**VDLLK-*OH*

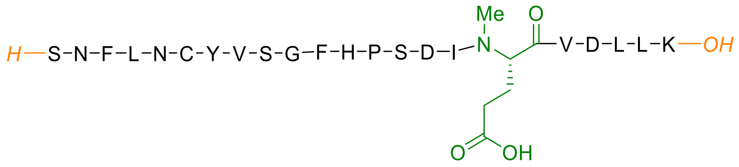

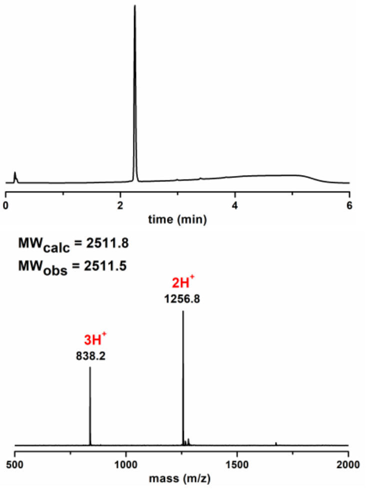

The isolated yield: 96 mg (38% based on the resin loading).

peptide F:

N-methylated amino acids: ***N*-Me-Glu17**

sequence:

Thz-GGG-SNFLN***Orn(linker)***YVSGFHPSDI***N*-Me-Glu**VDLLK-*OH*

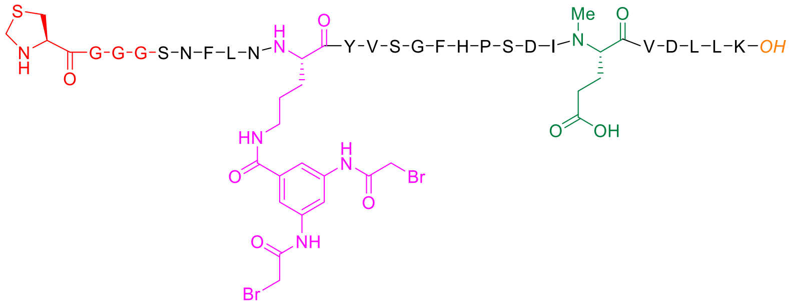

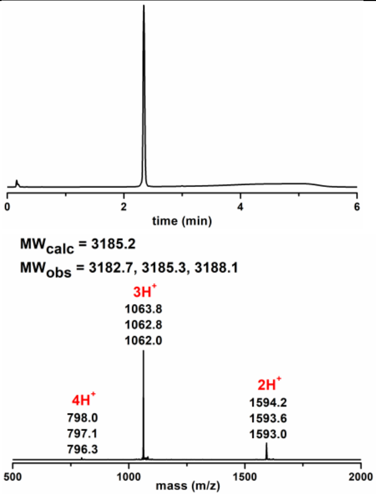

The isolated yield: 85 mg (30% based on the resin loading).

Table S2. Building blocks used to assemble **TofT-2** construct

	Building block	***Analytical characterization***
Covalent trimers:		
**NMT-A-2**	*N*-methylated amino acids: ***N*-Me-Asn5, *N*-Me-Val18** 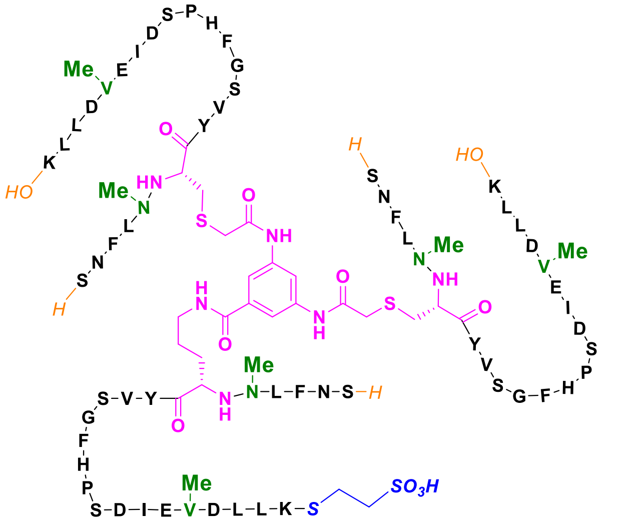	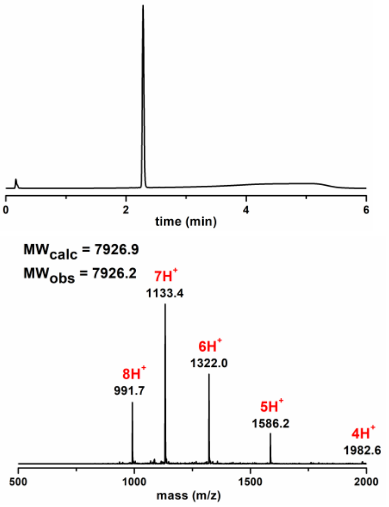 The isolated yield: 27.1 mg (49%).
**CT-1**	Same as in the synthesis of **TofT-1 (see Table S1)**	
**NMT-B-2**	*N*-methylated amino acids: ***N*-Me-Leu4, *N*-Me-Asp19** 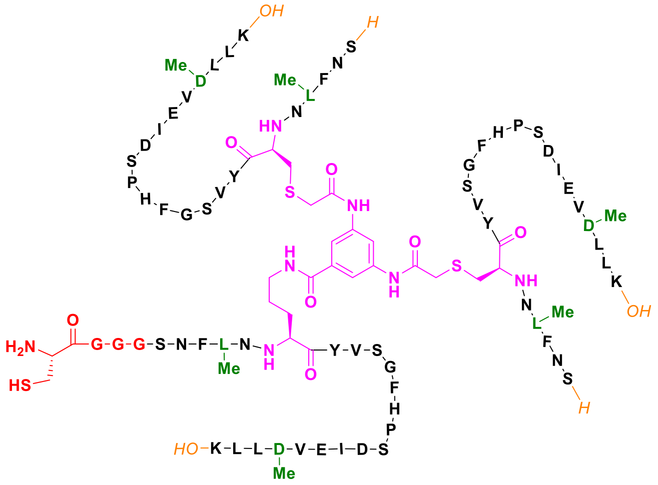	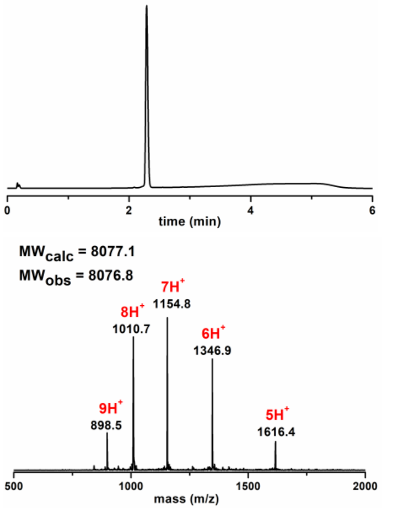 The isolated yield: 36.8 mg (66%).

Peptides used in the synthesis of covalent trimers:

peptide C: same as in the synthesis of **TofT-1** (see Table S1)

peptide D: same as in the synthesis of **TofT-1** (see Table S1)

Continued on p. 16

Continued from p. 15

peptide G:

N-methylated amino acids: ***N*-Me-Asn5, *N*-Me-Val18**

sequence:

H-SNFL***N*-Me-AsnCys**YVSGFHPSDIE***N*-Me-Val**DLLK-*OH*

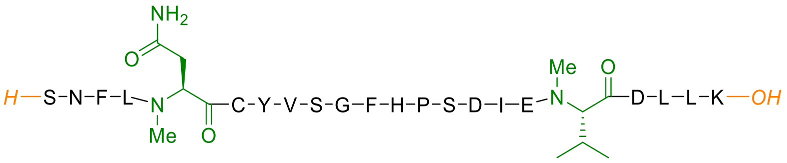

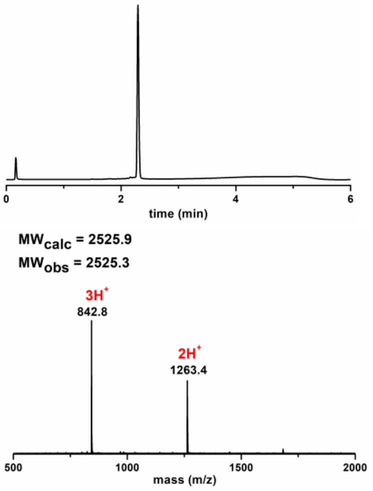

The isolated yield: 76 mg (30% based on the resin loading).

peptide H:

N-methylated amino acids: ***N*-Me-Asn5, *N*-Me-Val18**

sequence:

H-SNFL***N*-Me-Asn*Orn(linker)***YVSGFHPSDIE***N*-Me-Val**DLLK-***NHNH$_2$***

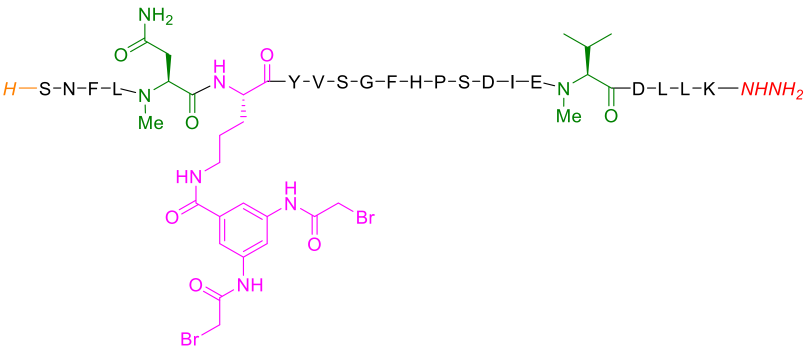

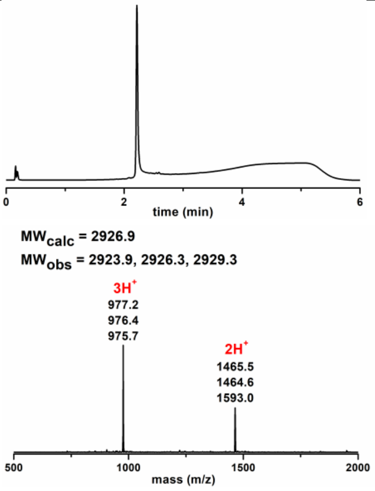

The isolated yield: 150 mg (33% based on the resin loading).

peptide I:

N-methylated amino acids: ***N*-Me-Leu4, *N*-Me-Asp19**

sequence:

H-SNF***N*-Me-Leu**N**Cys**YVSGFHPSDIEV***N*-Me-Asp**LLK-*OH*

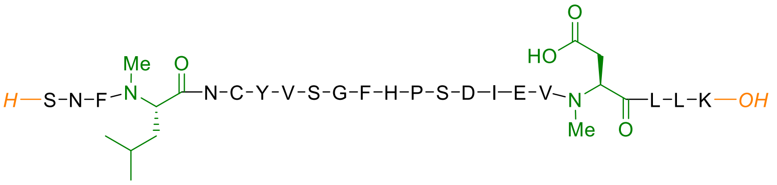

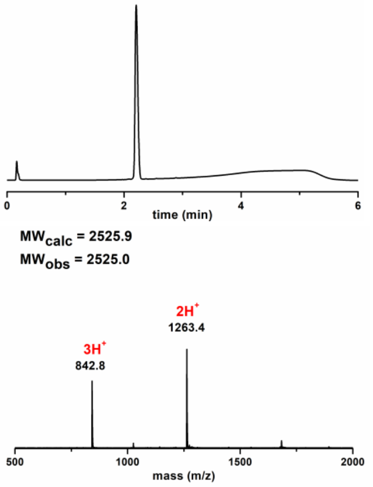

The isolated yield: 77 mg (30% based on the resin loading).

Continued on p. 17

Continued from p. 16

peptide J:

N-methylated amino acids: ***N*-Me-Leu4, *N*-Me-Asp19**

<u>*sequence:*</u>

Thz-GGG-SNF***N*-Me-Leu**N***Orn(linker)***YVSGFHPSDIEV***N*-Me-Asp**LLK-*OH*

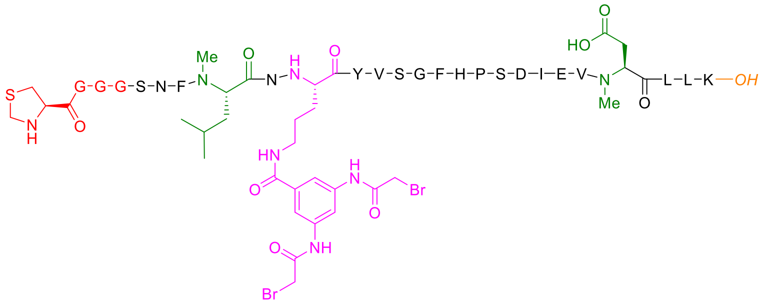

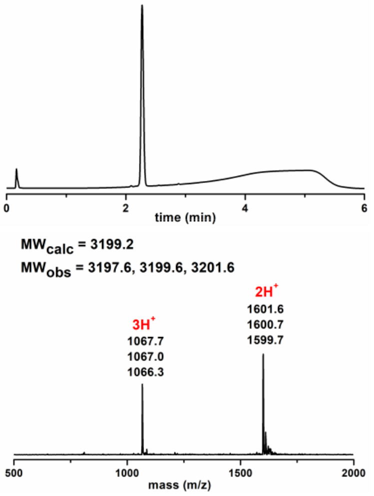

The isolated yield: 71 mg (22% based on the resin loading).

Table S3. Building blocks used to assemble **TofT-3** construct

	Building block	***Analytical characterization***
Covalent trimers:		
**NMT-A-2**	Same as in the synthesis of **TofT-2** (see Table S2)	
**CT-2**	^{15}N-labeled amino acids: **Phe3, Val8, Gly10, Ile16, Leu20**<br>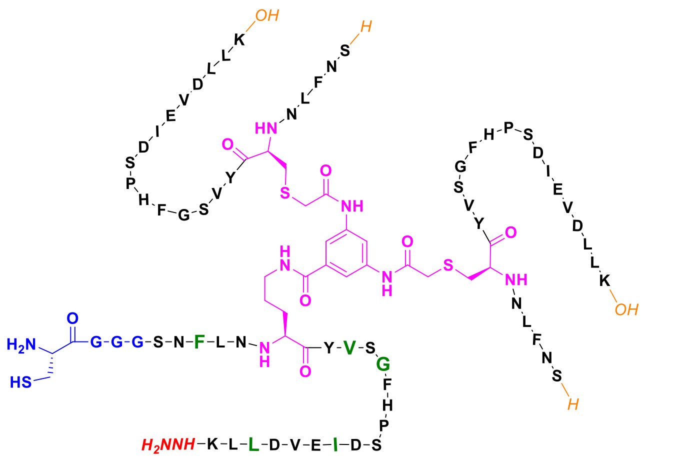	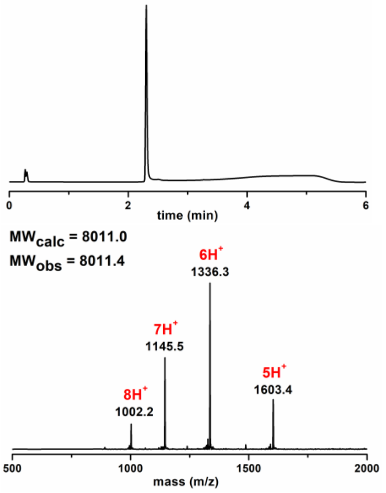<br>The isolated yield: 19.2 mg (35%).
**NMT-B-2**	Same as in the synthesis of **TofT-2** (see Table S2)	
Peptides used in the synthesis of covalent trimers:		
**peptide C:**	same as in the synthesis of **TofT-1** (see Table S1)	
**peptide K:**	^{15}N-labeled amino acids: **Phe3, Val8, Gly10, Ile16, Leu20**<br><u>*sequence:*</u> **Thz-GGG**-SN**F**LN***Orn(linker)***Y**V**S**G**FHPSD**I**EVD**L**LK-***NHNH$_2$***<br>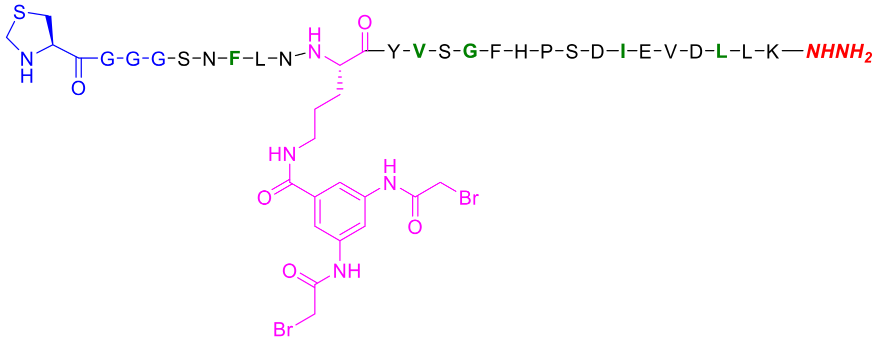	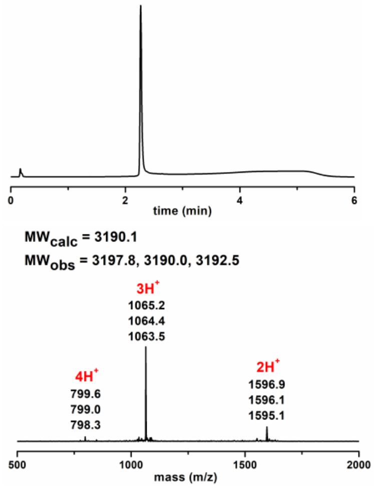<br>The isolated yield: 54 mg (17% based on the resin loading).
**peptide G**:	Same as in the synthesis of **TofT-2** (see Table S2)	
**peptide H:**	Same as in the synthesis of **TofT-2** (see Table S2)	
**peptide I:**	Same as in the synthesis of **TofT-2** (see Table S2)	
**peptide J:**	Same as in the synthesis of **TofT-2** (see Table S2)	

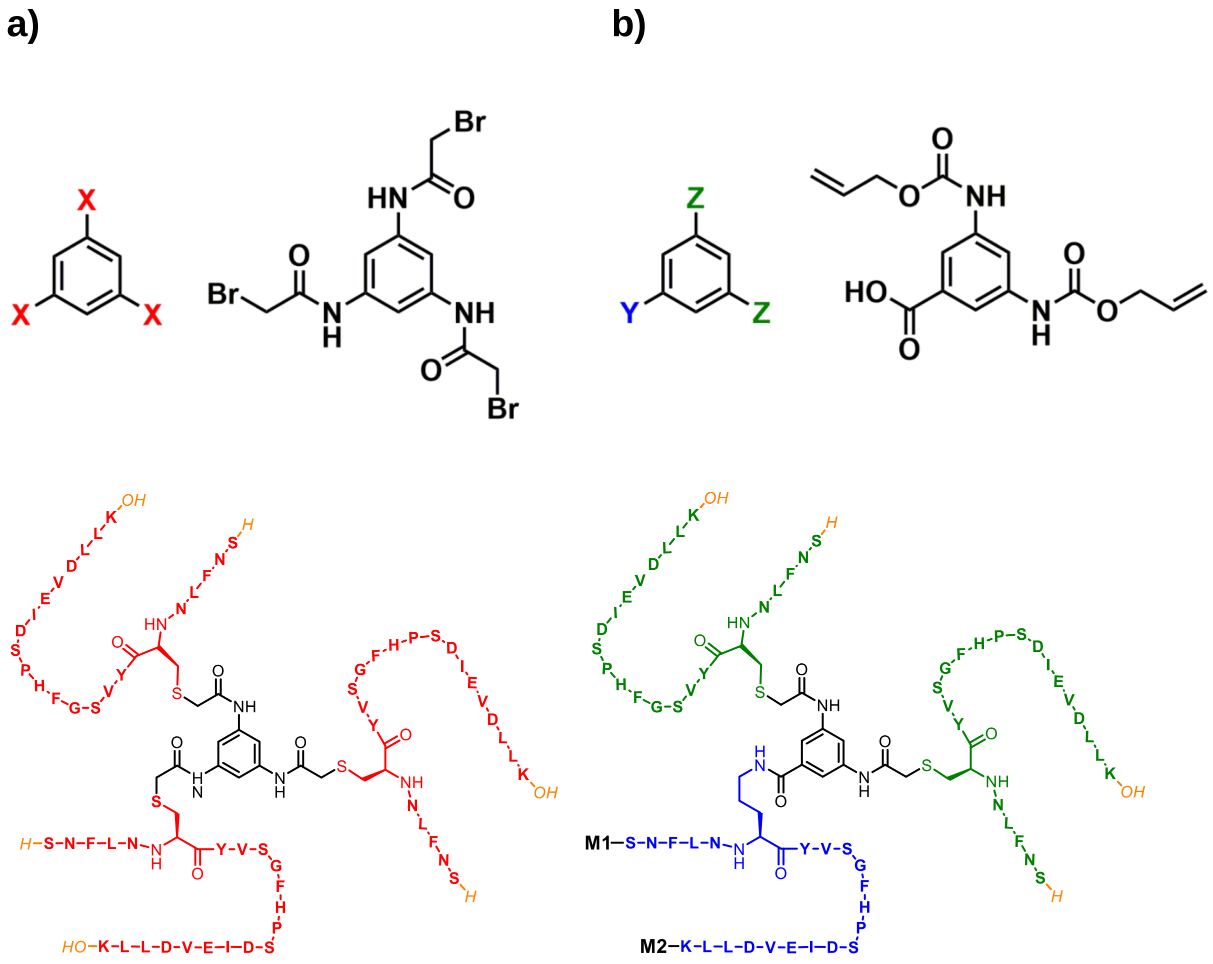

Fig. S1. Chemical structures of the central linkers. a) The linker used for the formation of symmetric trimers of [20-41]β2m peptide ("*covalent trimer*").(8) b) The new central linker that allows for synthesis of covalent trimers with non-identical peptides attached, M1 = modification 1 and M2 = modification 2. '*H*' and '*OH*' in orange designate unmodified amino and carboxy functions of *N*- and *C*-termini of peptide chain, respectively.

a)

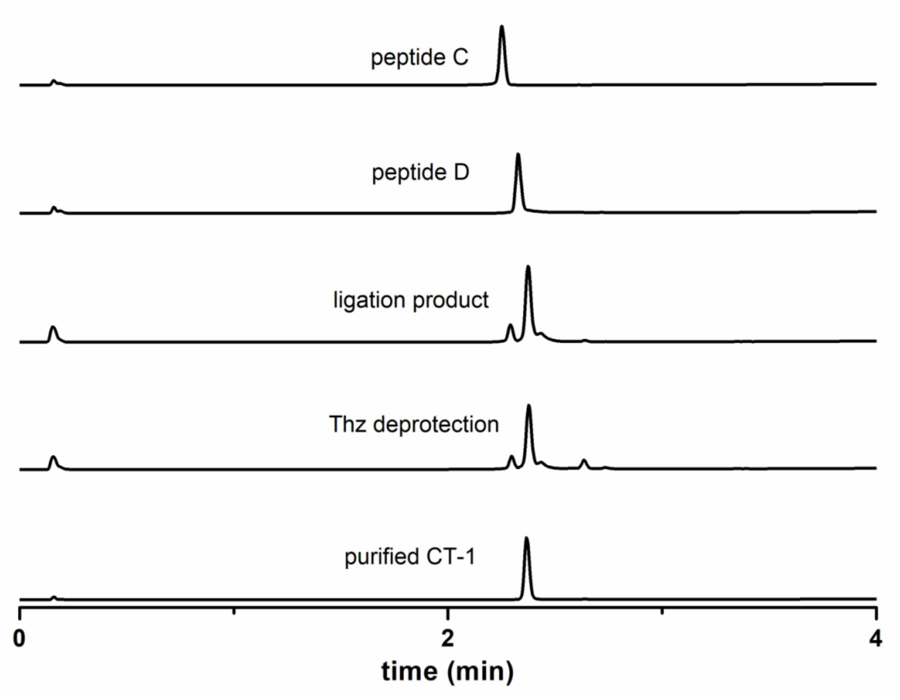

b)

Deconvoluted MS of the reaction between **peptides C** and **D**:

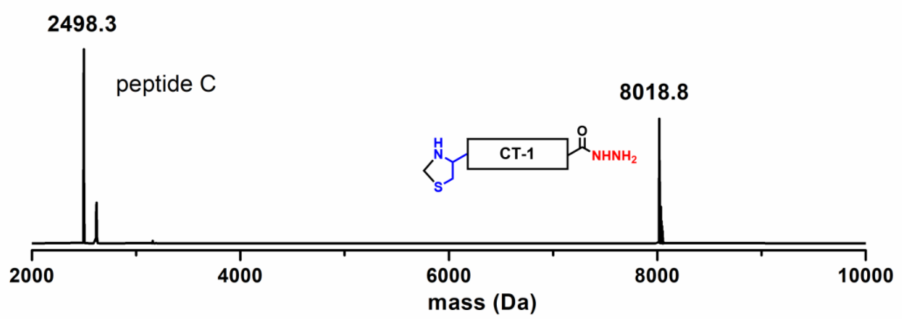

Deconvoluted MS after the conversion of Thz (thiazolidine) into Cys:

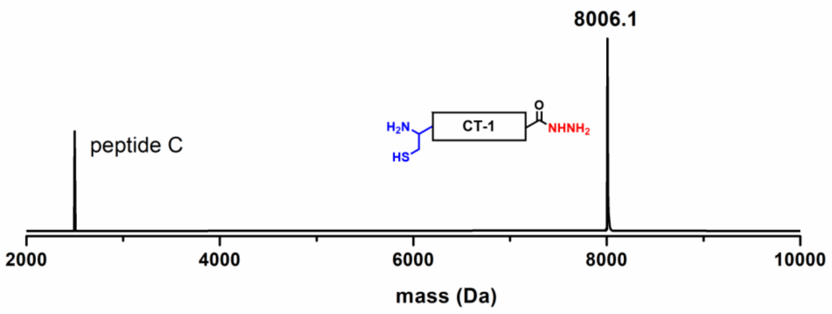

Fig. S2. a) Analytical HPLC and b) LC-MS monitoring of the reaction steps in the synthesis of non-symmetric covalent trimer **CT-1** (see Table S1).

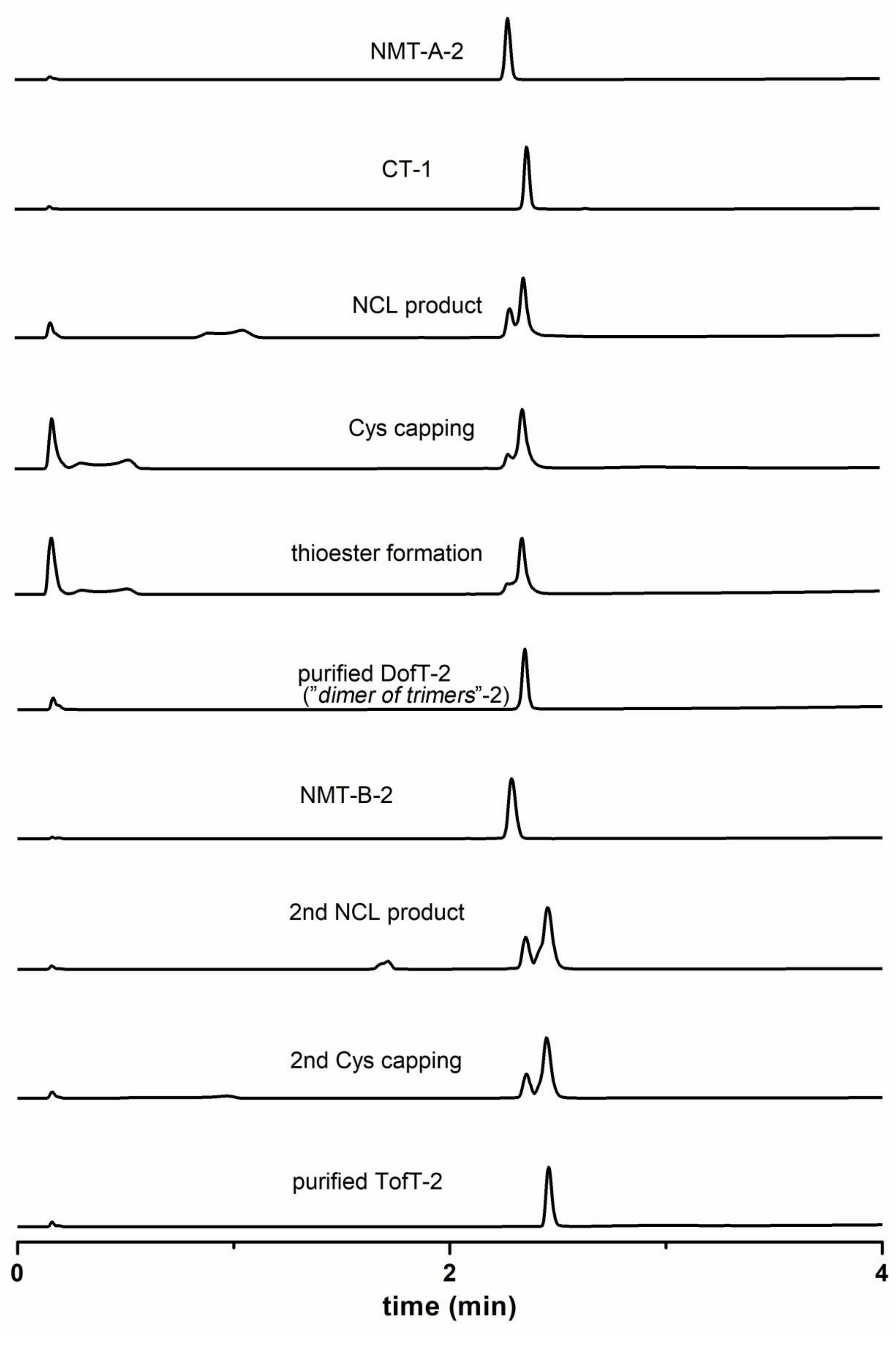

Fig. S3 (*continued on p. 22*). Analytical HPLC and LC-MS monitoring of the reaction steps in the sequential native chemical ligation in the total synthesis of **TofT-2** construct.

Deconvoluted MS after the native chemical ligation between **NMT-A-1** and **CT-1**:

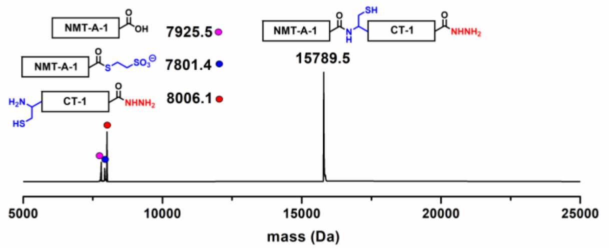

Deconvoluted MS after Cys capping at the ligation site:

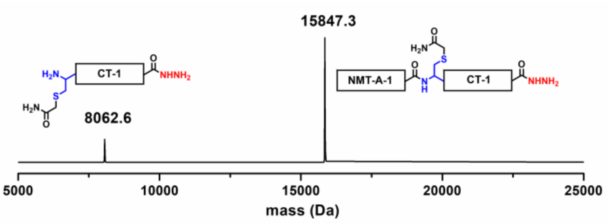

Deconvoluted MS after conversion of peptide-$^{\alpha}$hydrazide into peptide-$^{\alpha}$thioester:

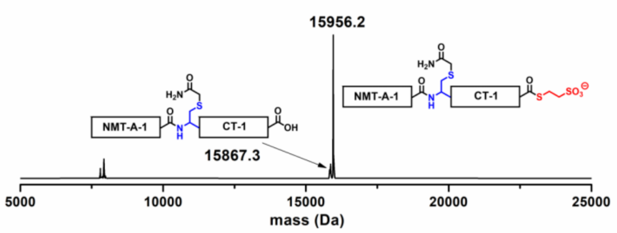

Deconvoluted MS after native chemical ligation between **DofT(“*dimer of trimers*”)-2** and **NMT-A-2**:

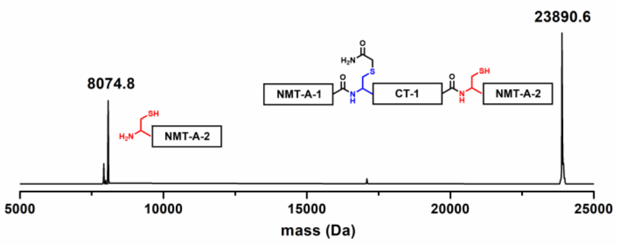

Deconvoluted MS after Cys capping at the ligation site:

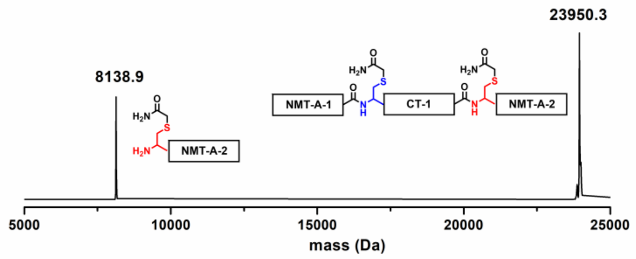

Fig. S3 (*continued from p. 21*). Analytical HPLC and LC-MS monitoring of the reaction steps in the sequential native chemical ligation in the total synthesis of **TofT-2** construct.

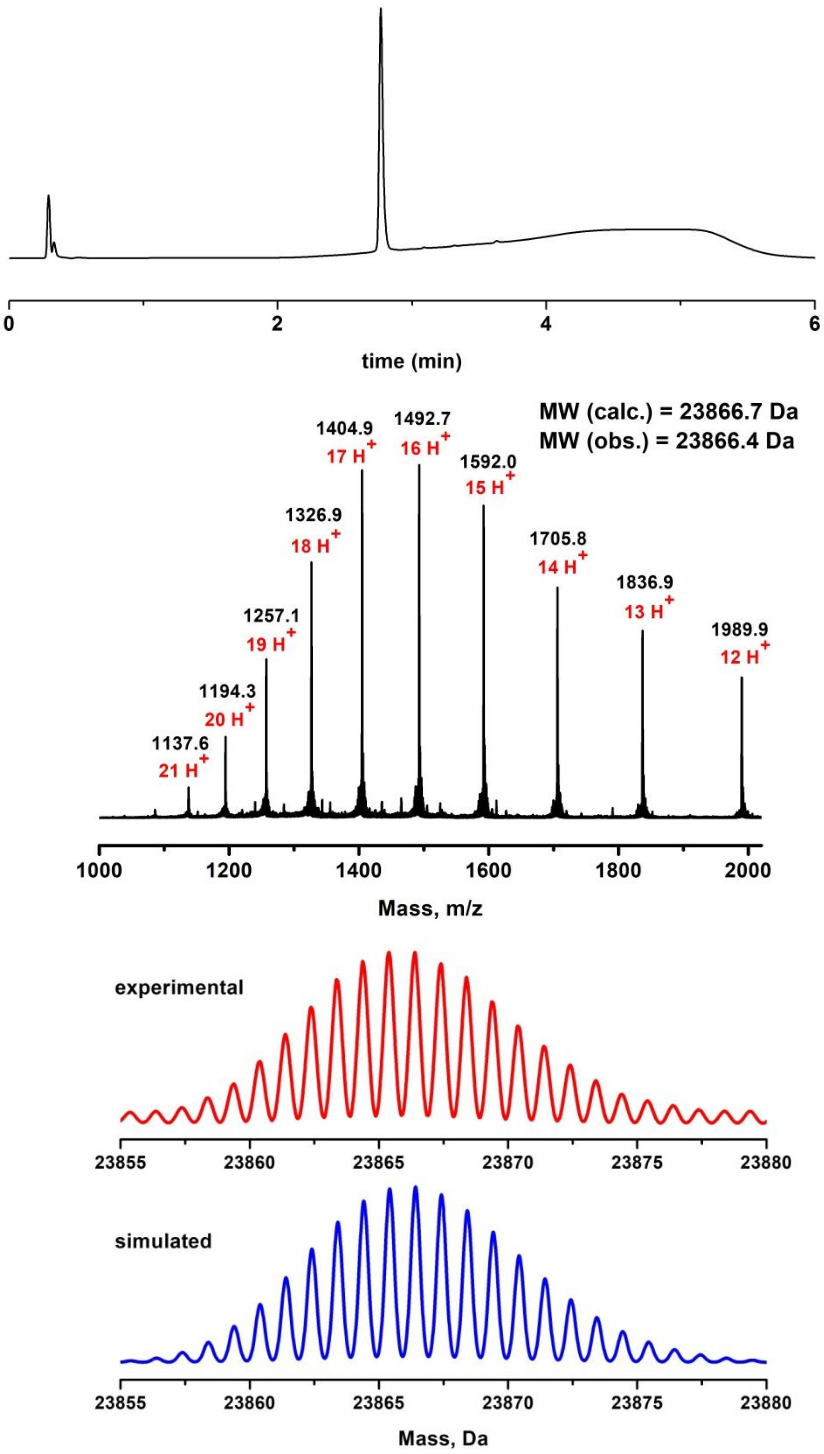

Fig. S4. Characterization of **TofT-1**. Top: Analytical HPLC (λ=220 nm); Middle: ESI-MS-Orbitrap mass spectrum; Bottom: Deconvolution of the experimental mass spectrum in red and theoretical simulation in blue.

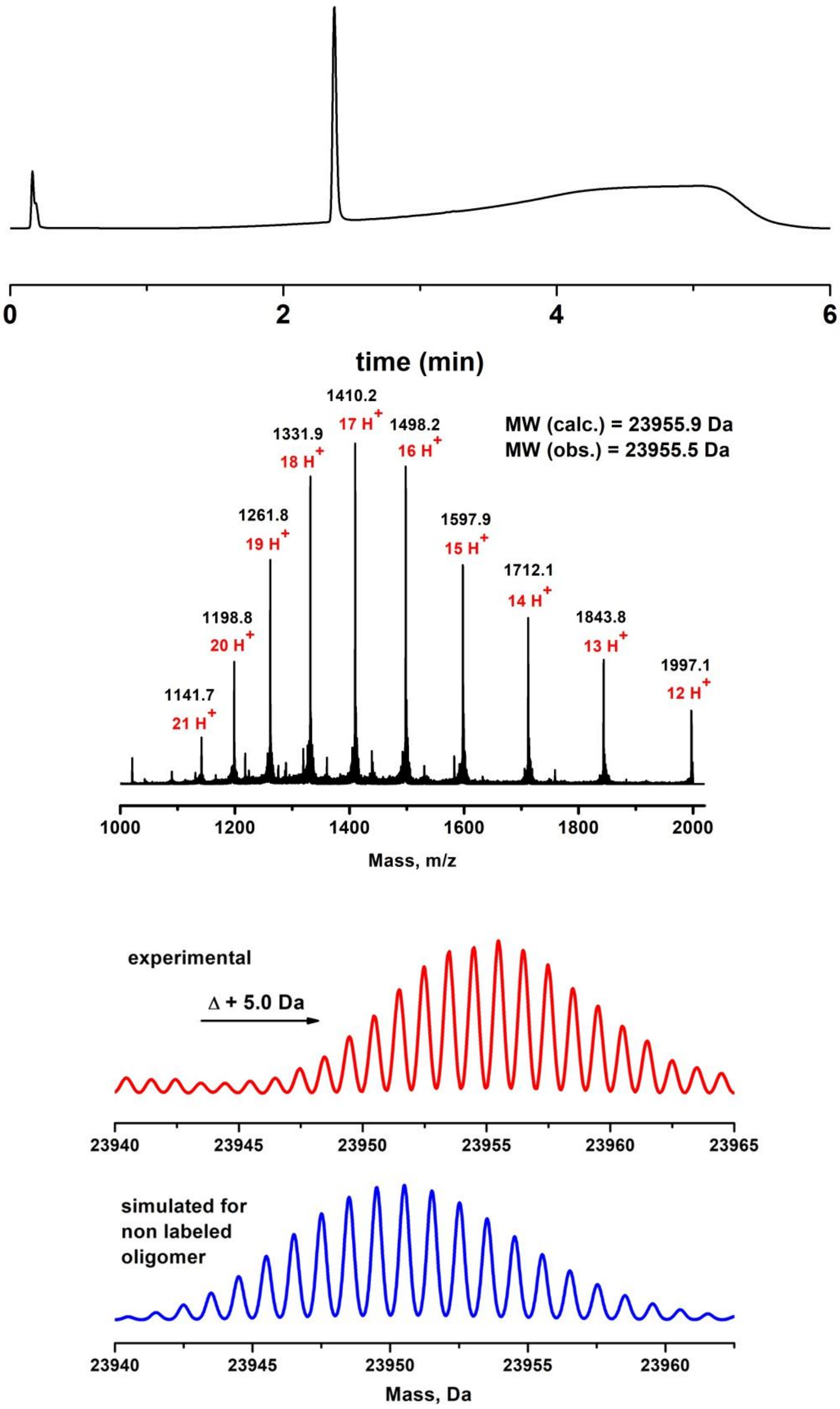

Fig. S5. Characterization of **TofT-3**. Top: Analytical HPLC (λ=220 nm); Middle: ESI-MS-Orbitrap mass spectrum; Bottom: Deconvolution of the experimental mass spectrum in red and theoretical simulation in blue.

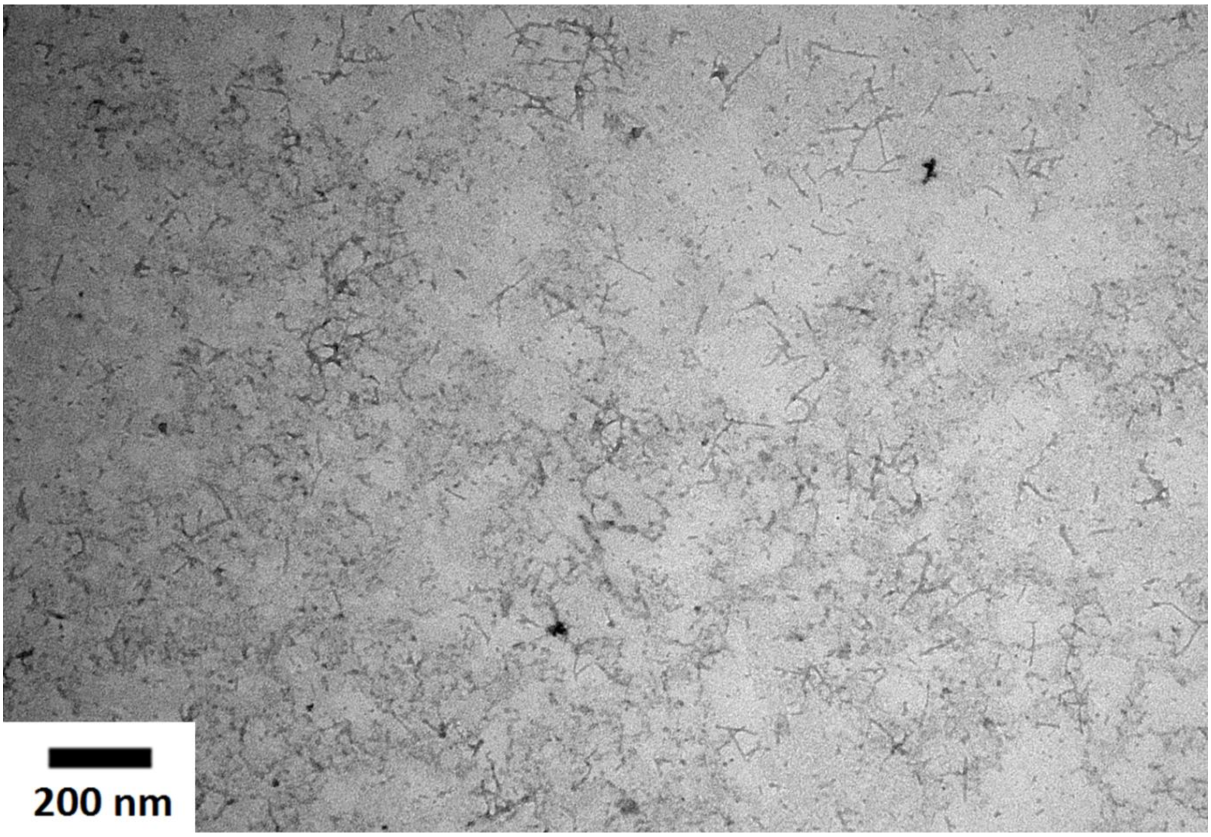

Fig. S6. TEM image of short fibers observed in **TofT-1** sample.

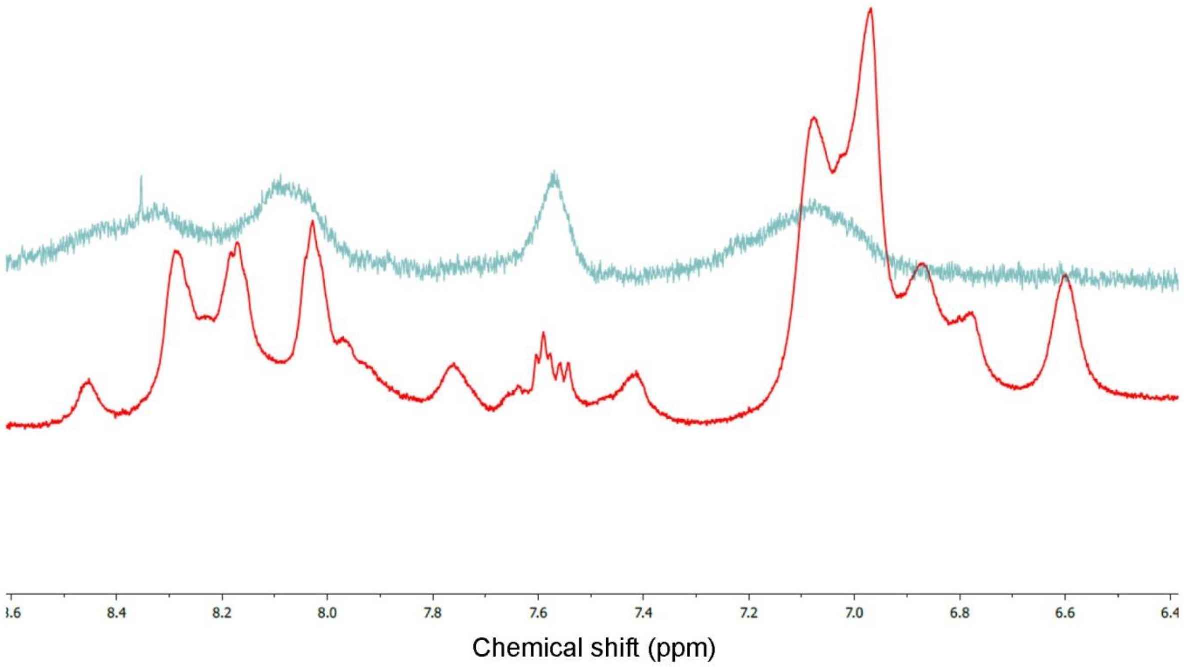

Fig. S7. ^{1}H NMR (amide and aromatic region) of **TofT-1** (in cyan) showing only broad peaks of peptide construct indicating an extensive aggregation. ^{1}H NMR of **TofT-2** (in red) shows well dispersed peaks coming from peptide construct, which indicates smaller size and high solubility of the species.

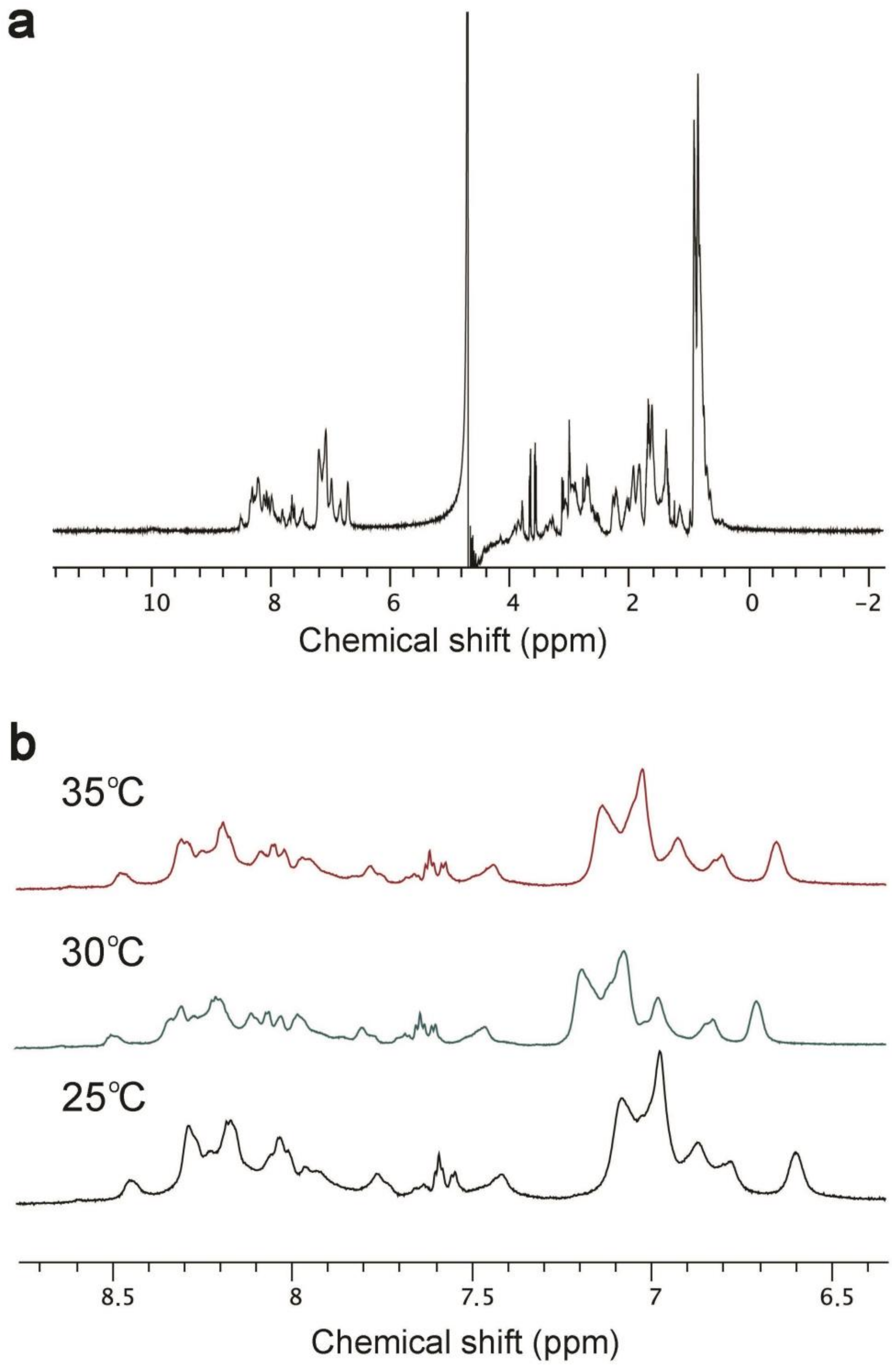

Fig. S8. a) ^{1}H NMR of **TofT-3** (200 µM in 50 mM phosphate buffer, pH 7) at 35 °C. b) Temperature dependence of amide and aromatic region of the ^{1}H NMR spectrum of **TofT-3** recorded at 25, 30 and 35 ºC.

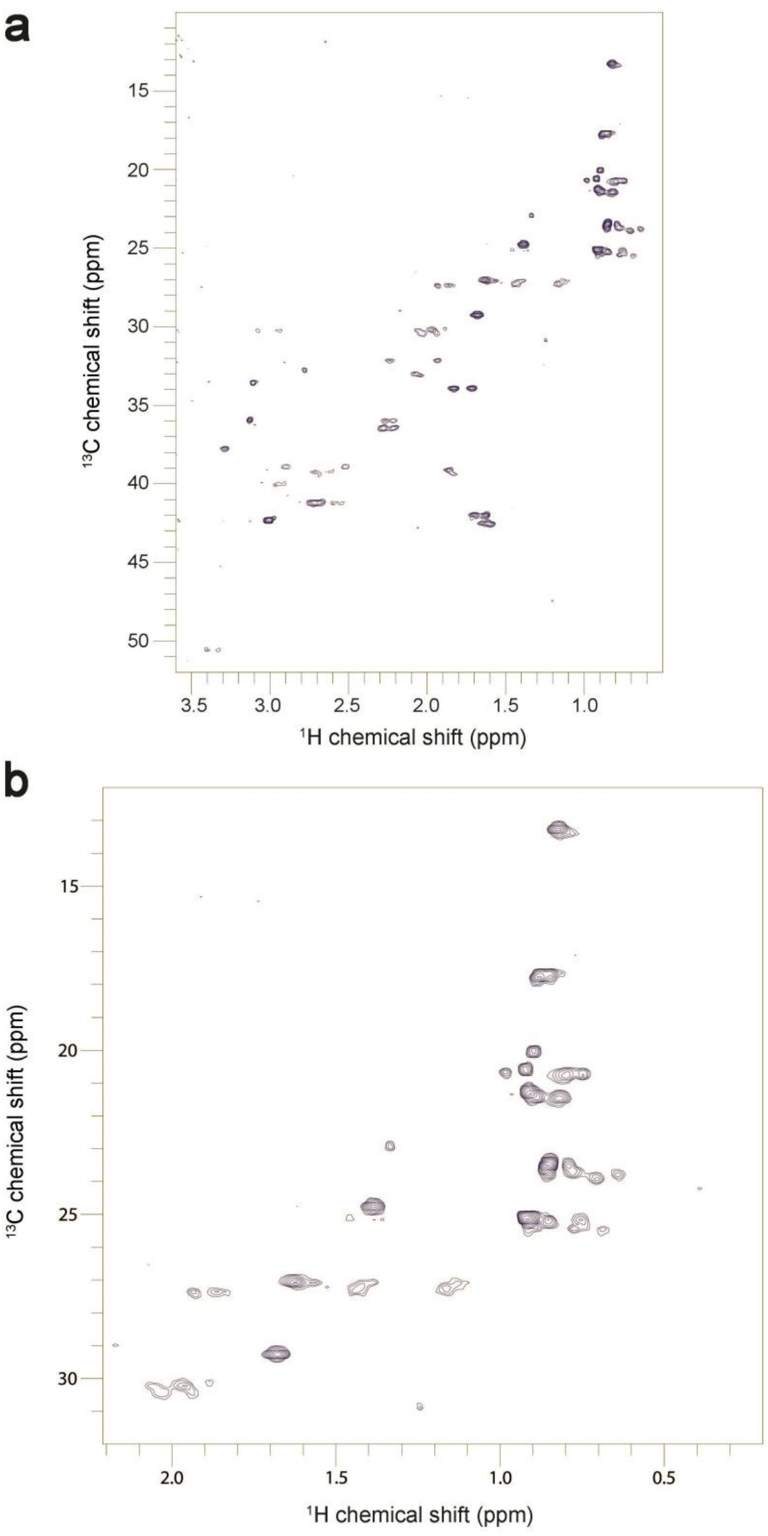

Fig. S9. a) ^{1}H-^{13}C HSQC NMR spectrum of **TofT-3**. b) Part in the spectrum that corresponds to methyl groups. Methyl resonances of different intensities suggest the presence of regions that differ in their conformational dynamics.

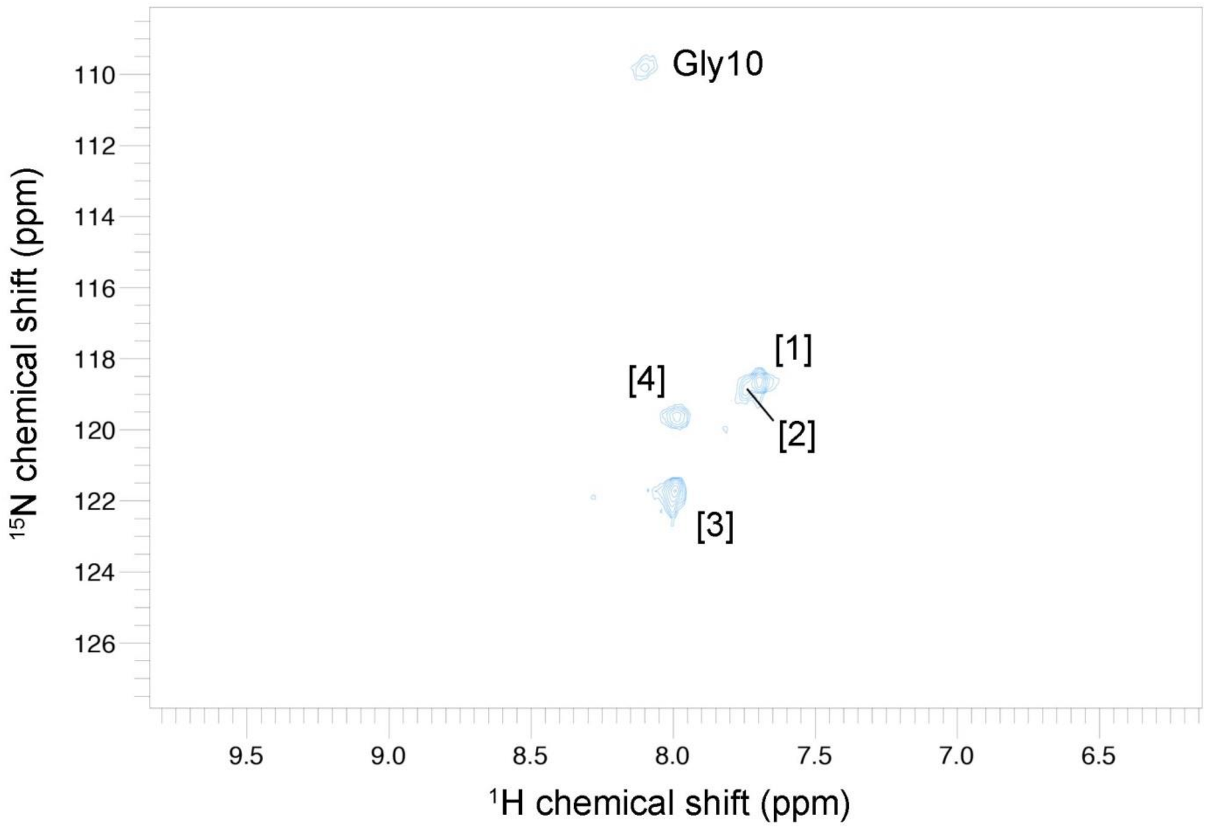

Fig. S10. ^{1}H-^{15}N HSQC spectrum of ^{15}N-labeled **TofT-3** construct at 35 °C.

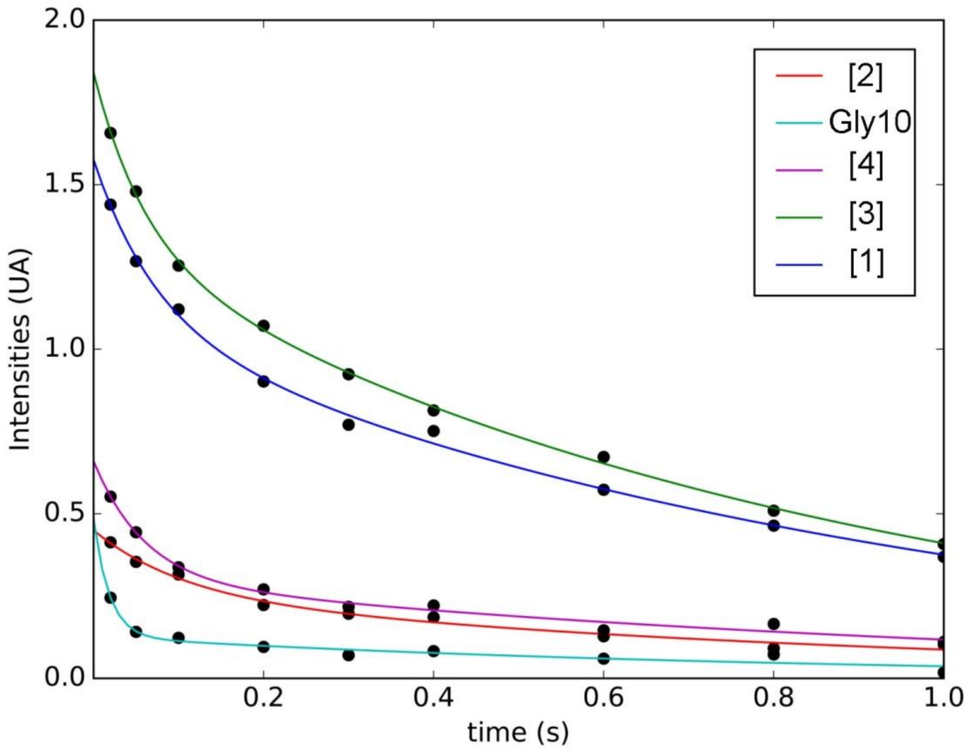


```
[1]     T1 69.1% :  938.7 ± 76.1 ms    T1 30.9% :   73.1 ± 19.3 ms
[2]     T1 56.6% :  934.7 ± 280.1 ms   T1 43.4% :  104.8 ± 50.0 ms
[3]     T1 71.3% :  860.7 ± 31.7 ms    T1 28.7% :   60.4 ± 9.1 ms
[4]     T1 45.5% :  1067.9 ± 223.1 ms  T1 54.5% :   59.9 ± 15.7 ms
[Gly10] T1 26.1% :  810.1 ± 250.9 ms   T1 73.9% :   18.5 ± 10.7 ms
```

Fig. S11. T_1 relaxation times of **TofT-3** measured at 35 ºC. The T_1 relaxation curves display clear bi-exponential decay behavior with a contribution of a very short T_1 (in the range of 20 to 100 ms) and a contribution of a long T_1 (in the range of 1 s). The occurrence of a fast component for the T_1 relaxation suggests the presence of a supplementary relaxation mechanism, in addition to the dipolar and chemical shift anisotropy mechanisms that usually dominate the relaxation of an isolated ^{15}N. A possible explanation is the occurrence of a slow exchange between soluble **TofT-3** and minute amounts of high molecular weight aggregates with the rate slower than the T_2 (100 ms).

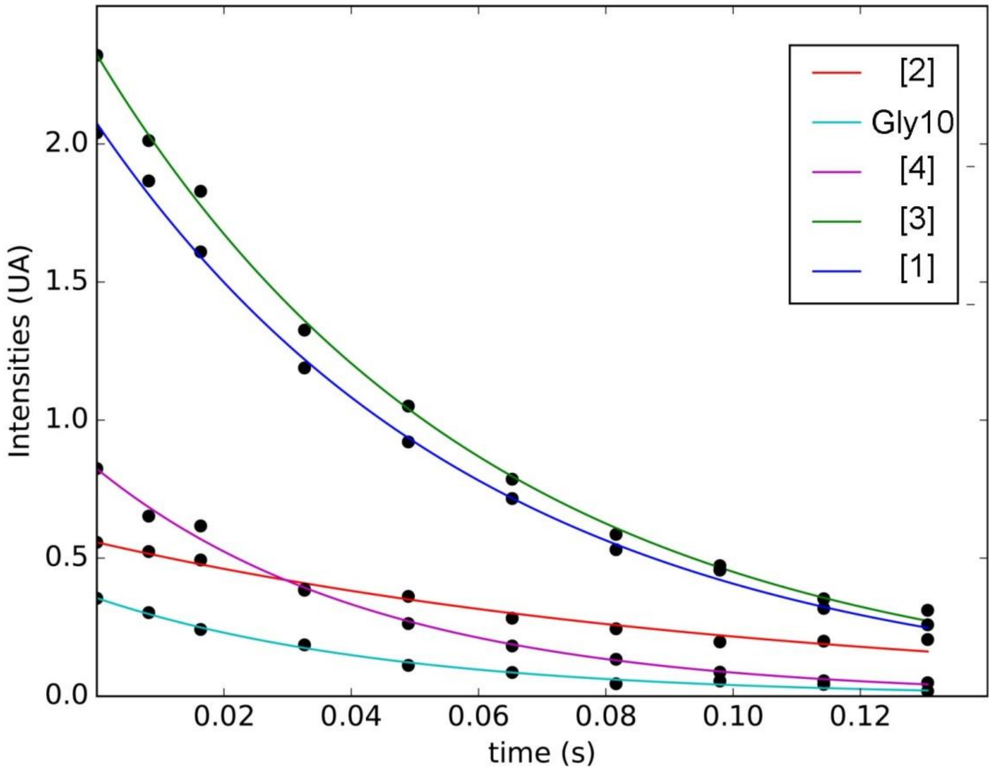


```
[1] T2   61.5 ± 1.2 ms
[2] T2  105.7 ± 6.5 ms
[3] T2   61.0 ± 1.1 ms
[4] T2   44.2 ± 1.7 ms
[Gly10] T2   46.0 ± 2.0 ms
```

Fig. S12. The T_2 relaxation curves displayed expected mono-exponential decays for all five resonances, including the low intensity GLY10. Most amide nitrogens display fast T_2 relaxation times between 40 and 60 ms, in agreement with homogeneous dynamic behavior of the construct. Only the peak [2] is characterized by a longer T_2, suggesting a faster correlation time for this site.

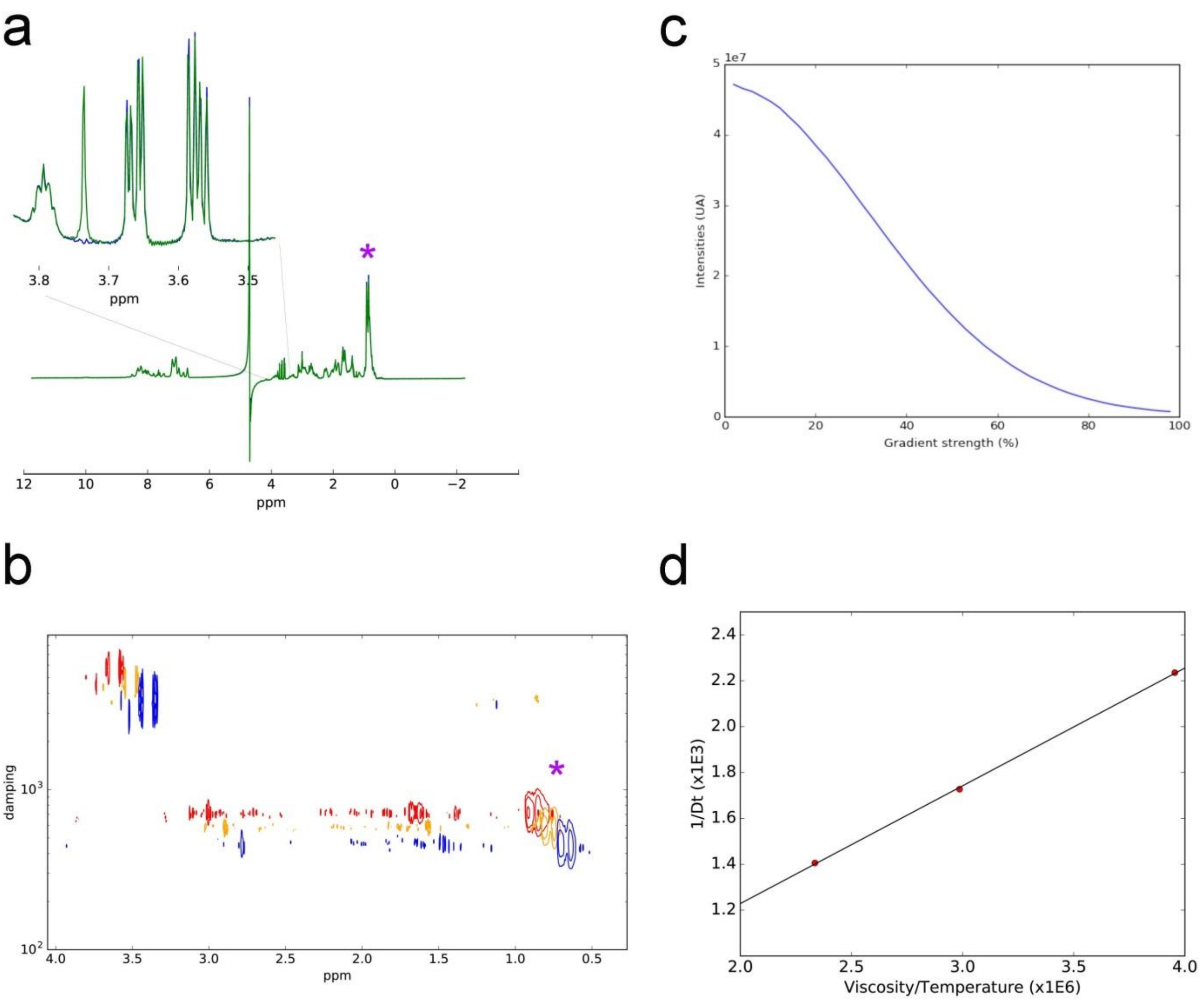

Ratios of the TRIS/**TofT-3** translational diffusion coefficients:

35 °C (308 K) 0.158
25 °C (298 K) 0.165
15 °C (288 K) 0.153

Fig. S13. DOSY measurements. a) Spectrum of **TofT-3** without (blue) and with TRIS (tris(hydroxymethyl)aminomethane) added (green). b) DOSY spectra recorded at temperatures of 15 °C (blue), 25 °C (orange) and 35 °C (red). The asterisk in purple indicates the methyl peaks used for calculation of diffusion coefficient. c) The diffusion coefficients were obtained after the Laplace transform of the DOSY spectra using the PALMA server (https://arxiv.org/abs/1608.07055). d) As a function of temperature, the diffusion coefficient displays the expected linear dependence when corrected to the variation of viscosity and corresponds to molecular weight of ~20 kDa.[5]

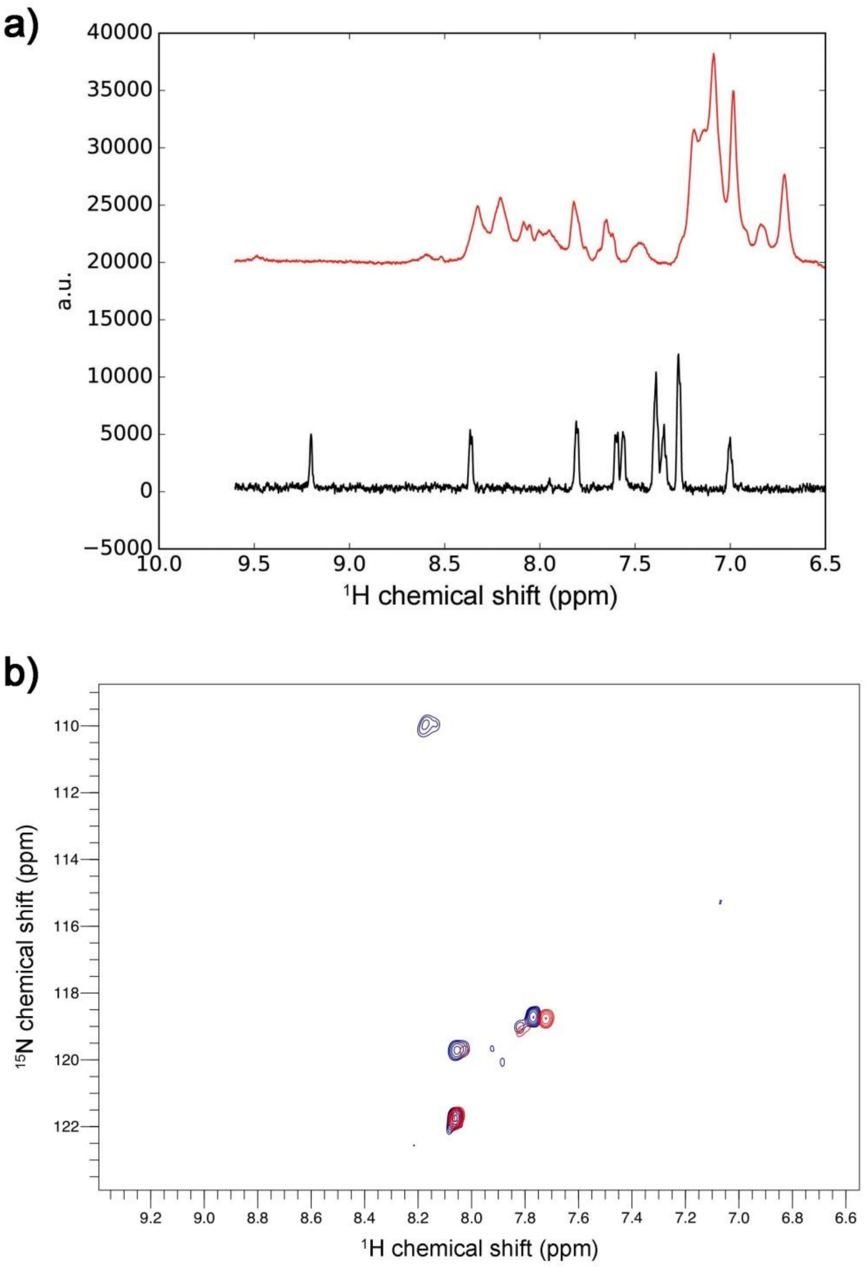

Fig. S14. Binding of bis-ANS to **TofT-3** was directly verified by solution NMR. a) ^{1}H NMR spectra of bis-ANS in the presence of equimolar quantities **TofT-3** (in red) and free bis-ANS (in black). b) Overlay of ^{1}H-^{15}N HSQC spectra of **TofT-3** (in blue) and upon addition of bis-ANS (in red).

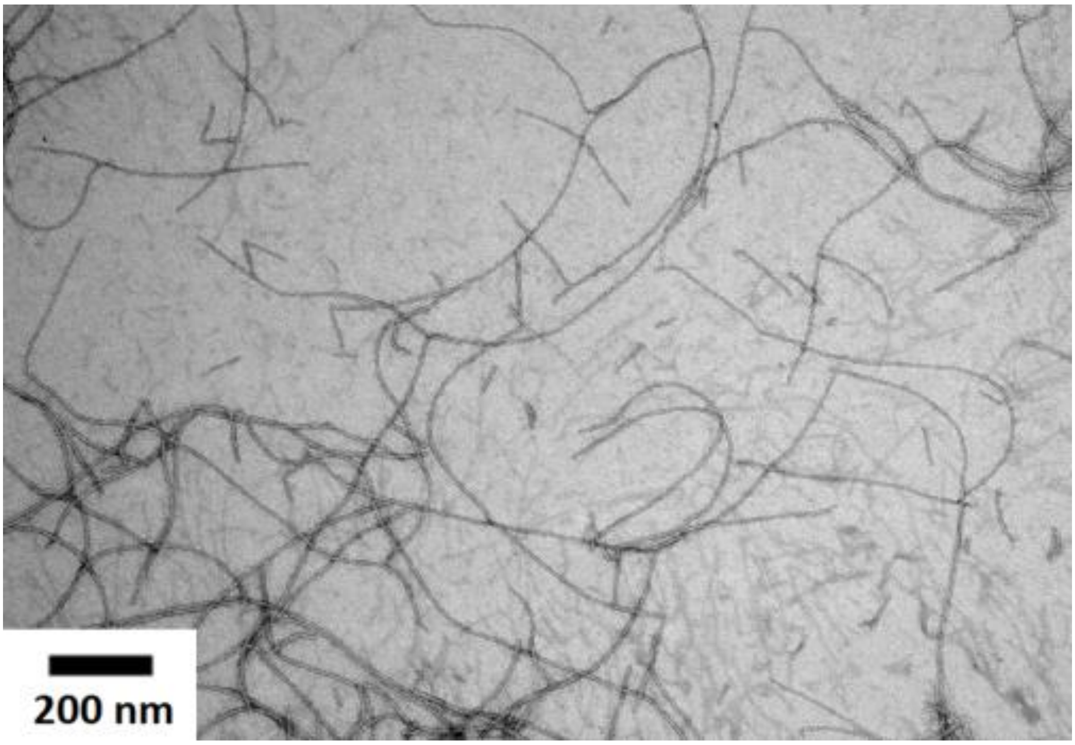

Fig. S15. TEM image of amyloid fibers obtained from central trimer **CT** have elongated morphology similar to “covalent trimer” [20-41]β2m amyloids.[(8)] This also indicates that the structures of the central linker (in **CT** it is different from “covalent trimer” [20-41]β2m, see Figure S1) are not detrimental in the amyloid self-assembly mechanism.

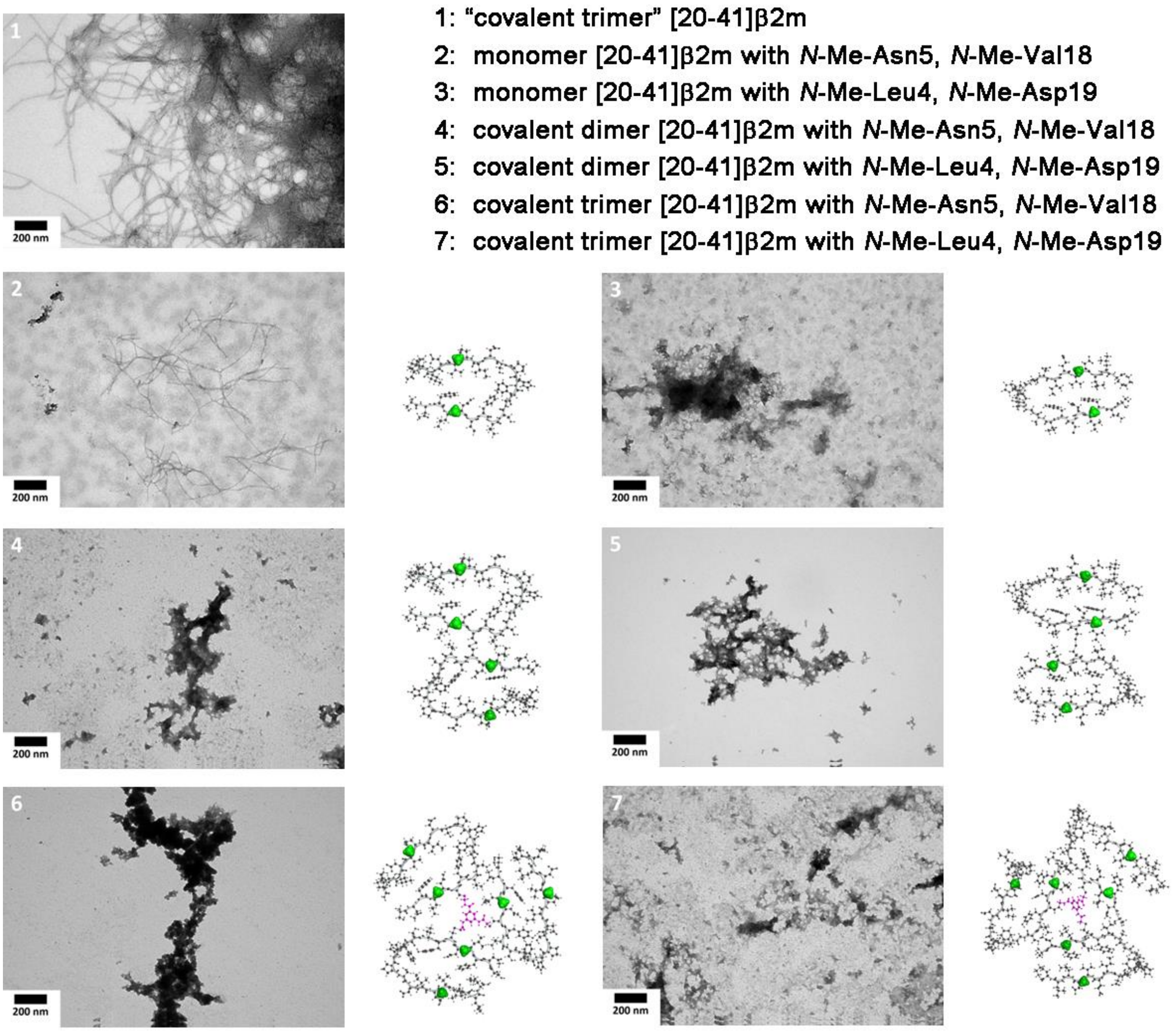

Fig. S16. Transmission electron microscopy (TEM) analysis of the aggregates obtained upon completion of the experiment described in Figure 5 in the main manuscript. *Panel 1* depicts the long fibers observed for the *"covalent trimer"* [20-41]β2m (reference construct). *Panels 2-7* show morphologies of different aggregates obtained in the presence of *N*-methylated peptide inhibitors (their molecular structures are shown to the right of the corresponding TEM images). In the presence of covalent dimers and trimers, the fibrillar morphology was not observed indicating their higher potency to modify the amyloid self-assembly.

References:

(1) N. A. Farrow, R. Muhandiram, A. U. Singer, S. M. Pascal, C. M. Kay, G. Gish, S. E. Shoelson, T. Pawson, J. D. Forman-Kay, L. E. Kay, Backbone dynamics of a free and phosphopeptide-complexed Src homology 2 domain studied by ^{15}N NMR relaxation. *Biochemistry* 1994, **33**, 5984-6003.

(2) F. Delaglio, S. Grzesiek, G. W. Vuister, G. Zhu, J. Pfeifer, A. Bax, NMRPipe: a multidimensional spectral processing system based on UNIX pipes. *J. Biomol. NMR* 1995, **6**, 277-293.

(3) W. F. Vranken, W. Boucher, T. J. Stevens, R. H. Fogh, A. Pajon, M. Llinas, E. L. Ulrich, J. L. Markley, J. Ionides, E. D. Laue, The CCPN data model for NMR spectroscopy: development of a software pipeline. *Proteins* 2005, **59**, 687-696.

(4) V. Daragan, K. Mayo, Motional model analyses of protein and peptide dynamics using C-13 and N-15 NMR relaxation. *Prog. Nucl. Magn. Res. Spectrosc.* 1997, **31**, 63–105.

(5) S. Augé, P. O. Schmit, C. A. Crutchfield, M. T. Islam, D. J. Harris, E. Durand, M. Clemancey, A. A. Quoineaud, J. M. Lancelin, Y. Prigent, F. Taulelle, M. A. Delsuc, NMR measure of translational diffusion and fractal dimension. Application to molecular mass measurement. *J. Phys. Chem. B* 2009, **113**, 1914-1918.

(6) MOE: Molecular Operating Environment (MOE) software, v. 2015.10; Chemical Computing Group Inc., Montreal, Canada; http://www.chemcomp/MOE2015.htm.

(7) G.-M. Fang, J.-X. Wang, L. Liu, Convergent chemical synthesis of proteins by ligation of peptide hydrazides. *Angew. Chem. Int. Ed.* 2012, **51**, 10347–10350.

(8) J. Ruiz, R. Boehringer, M. Grogg, J. Raya, A. Schirer, C. Crucifix, P. Hellwig, P. Schultz, V. Torbeev. Covalent tethering and residues with bulky hydrophobic side chains enable self-assembly of distinct amyloid structures. *ChemBioChem* 2016, **17**, 2274-2285.
